# Emerging evidence of microbial infection in causing systematic immune vasculitis in Kawasaki disease

**DOI:** 10.3389/fmicb.2023.1313838

**Published:** 2023-12-22

**Authors:** Wang Wang, Liyan Zhu, Xuan Li, Zhiheng Liu, Haitao Lv, Guanghui Qian

**Affiliations:** ^1^Institute of Pediatric Research, Children's Hospital of Soochow University, Suzhou, Jiangsu, China; ^2^Department of Experimental Center, Medical College of Soochow University, Suzhou, China; ^3^Department of Cardiology, Children's Hospital of Soochow University, Suzhou, Jiangsu, China

**Keywords:** bacteria, fungi, Kawasaki disease, vasculitis, virus

## Abstract

Kawasaki disease (KD) is a systematic vasculitis that is often complicated by coronary artery lesions and is a leading cause of acquired heart disease in developed countries. Previous studies have suggested that genetic susceptibility, together with an inducing infectious agent, could be involved in KD pathogenesis; however, the precise causative agent of this disease remains unknown. Moreover, there are still debates concerning whether KD is an infectious disease or an autoimmune disease, although many studies have begun to show that various pathogens functioning as critical inducers could activate different kinds of immune cells, consequently leading to the dysfunction of endothelial cells and systematic vasculitis. Here in this review, we attempt to summarize all the available evidence concerning pathogen infections associated with KD pathogenesis. We also discuss the related mechanisms, present a future perspective, and identify the open questions that remain to be investigated, thereby providing a comprehensive description of pathogen infections and their correlations with the host immune system in leading to KD.

## 1 Introduction

Pathogen infectious diseases have posed a great challenge to human health worldwide (Baldari et al., 2023). Currently, various pathogens have been suggested as critical triggers in inducing systematic vasculitis in children with Kawasaki disease (KD), which is a leading cause of acquired heart disease in developed countries (McCrindle et al., 2017). High-dose intravenous immunoglobulin (IVIG) infusion and aspirin can subdue KD symptoms and partially reduce the occurrence of coronary artery lesions (CALs); however, approximately 10%–20% of affected children develop recrudescent or persistent fever even after IVIG infusion, and those patients have a higher risk of CAL (Li et al., 2018; Nadig et al., 2023). Critically, if this disease is not untreated in a timely manner, sudden death may occur due to coronary artery aneurysms (Shulman and Rowley, 2015; McCrindle et al., 2017; Sosa et al., 2019). Although genetic background (Chang et al., 2014), urban industrialization, environmental factors (Chang et al., 2020; Corinaldesi et al., 2020), and regional winds together with large-scale atmospheric circulation (Rodo et al., 2011, 2014), have been suggested to correlate with KD, these theories fail to explain the seasonal epidemics of this illness, and also fail to explain why Kawasaki disease does not broadly recur. Nevertheless, an increasing number of epidemiological and clinical data all point to KD having an infectious etiology. For example, epidemiological data from multiple centers worldwide demonstrate that KD has a significant seasonal epidemic (Valtuille et al., 2023), frequent occurrence, and low recurrence characteristics in young children (Nakamura et al., 2008, 2012; Burns et al., 2013; Lin et al., 2015; Ozeki et al., 2017, 2018; Kido et al., 2019; Kim et al., 2020; Xie et al., 2020). Notably, several studies have shown that both the immune repertoire (Kuo et al., 2019) and the heterogeneous host immune response including the autoantibody responses in KD children resemble those observed in patients with bacterial or viral infections (Lindquist and Hicar, 2019; Jackson et al., 2021; Ghosh et al., 2022), lending further support of an infectious disease cause of KD.

Additionally, serum KD-specific molecules which were mostly derived from biofilms possessed molecular structures common to MAMPs (microbe-associated molecular pattern) from *Bacillus cereus*, *B. subtilis*, *Yersinia pseudotuberculosis* (*Y. pstb*), and *Staphylococcus aureus* (Kusuda et al., 2014), implicating a possible relationship between MAMPs and the etiological mechanism of KD vasculitis. Recently, at least 14 types of viruses have been suggested to correlate with KD based on serological and polymerase chain reaction (PCR) analysis of clinical samples (Principi et al., 2013). However, another study showed that at least 15 types of viruses were related to KD because the isolation rates of various viruses in KD patients were significantly higher than those in the control group (Huang et al., 2015; Jackson et al., 2021). Viral infections can cause vascular damage either through direct invasion of the vascular endothelium or provoking a rapid cell-damaging event (Hara et al., 2021). This in turn results in a larger release of proinflammatory cellular components from damaged endothelial cells, pyroptosis, or proinflammatory cell death (Mohandas et al., 2023), hence making various kinds of innate immune cells infiltrate the coronary arteries of KD subjects (Kuijpers et al., 1999; Takahashi et al., 2010a). These data thus suggest that different kinds of microbes are implicated in the pathogenesis of KD, but which microbes are the key inducers and the underlying mechanisms remain unclear. In this review, to better understand the comprehensive profiles between microbial infection and KD pathogenesis, we summarized the major features of our current understanding with respect to various pathogens related to KD. We also discuss the state of this field in KD with respect to the relationship and/or mechanisms concerning the abnormal immune response triggered by various infectious agents, and the open questions that remain to be investigated.

## 2 Involvement of pathogens during KD pathogenesis

### 2.1 Viral infection and KD

#### 2.1.1 DNA viruses

Several DNA viruses, including Epstein–Barr virus (EBV), human adenovirus, human parvovirus B19, torque teno virus, herpes family virus, varicella zoster virus, bocaparvo virus, and cytomegalovirus have been identified to be associated with KD pathogenesis.

##### 2.1.1.1 Human adenovirus

Adenovirus type 2 was first isolated from a patient with fatal Kawasaki disease (Embil et al., 1985), while another case report showed that human adenovirus infection can be found in monozygotic twin boys who developed KD (Fukuda et al., 2017). Among the adenovirus-infected cohort, the overall incidence of KD was 5.29 times higher than that of the non-adenovirus-infected control subjects (adjusted HR 5.29, 95% CI: 2.48–11.3), as shown by a population-based cohort study (Huang et al., 2020), suggesting a correlation between adenovirus infection and KD pathogenesis. Notably, there are also studies showing a lack of association between adenovirus infection and KD, suggesting that more intense research is needed to explore the relationships between adenovirus infections and KD (Okano et al., 1990; Shike et al., 2005).

##### 2.1.1.2 Human parvovirus B19

Human parvovirus B19 (HPV-B19) is a single-stranded DNA virus that may have a pathogenic role in the development of KD with other predisposing factors because it can cause symptoms resembling those observed in KD patients (Nigro et al., 1994; Holm et al., 1995). Importantly, HPV-B19 infection should be considered in the differential diagnosis of KD patients who show atypical clinical symptoms during the erythema infectiosum epidemic stage (Oura et al., 2022).

##### 2.1.1.3 Torque teno virus

The torque teno virus (TTV), which is a single-stranded circular DNA virus, was first found in the lymph node of a KD patient (Katano et al., 2012). For instance, a high viral load of torque teno virus 7 (TTV7) was identified in KD patients (Thissen et al., 2018; Spezia et al., 2023a), and the viral load of TTV positively correlated with the level of total bilirubin and aspartate aminotransferase in KD patients (Spezia et al., 2023b), suggesting that TTV might play a critical role in the pathophysiology of patients with KD.

##### 2.1.1.4 Herpes simplex virus

Herpes simplex virus (HSV) consists of multiple subtypes (Rowley et al., 2011), and its family members, including EBV, HHV-6 and varicella-zoster, were all found to be involved in KD. For instance, a previous study showed that the DNA sequence of EBV can be detected in KD patients (Kikuta et al., 1988), and there are many cases of KD-like lesions, specifically coronary artery aneurysms (CAAs), that were suggested to be caused by EBV infection (Kikuta et al., 1993; Rosenfeld et al., 2020; Xiao et al., 2020). However, EBV might not be the direct causative agent of KD, as shown by another study (Kikuta et al., 1990). Notably, a case of Kawasaki disease triggered by EBV virus infection was found to be complicated with familial Mediterranean fever (Maggio et al., 2019). Moreover, the prevalence of EBV in KD children was significantly lower during the early stage (van Stijn et al., 2020), and deoxyuridine 5′-triphosphate nucleotide hydrolase (dUTPase), a pathogen nonstructural protein encoded by EBV, can stimulate monocyte-derived macrophages through Toll-like receptor 2-dependent signaling transduction (Ariza et al., 2009), suggesting that DUTPase could be used as a potential target for drug development against EBV infection and KD treatment.

In addition to EBV, certain KD patients also have concomitant varicella zoster virus or coxsackievirus A4 infection (Turkay et al., 2006; Toprak et al., 2015). Given that the features of HHV6-infected patients resemble those symptoms observed in KD children (Kakisaka et al., 2012; Alramadhan et al., 2020), HHV-6B was thus suggested to be a critical mediator during the pathogenesis of KD, and HHV-6B infection was also suggested to be responsible for the increased number of KD patients during the SARS-CoV-2 pandemic (Dursun and Temiz, 2020).

##### 2.1.1.5 Bocavirus

Human bocavirus (HboV) is a single-stranded DNA etiologic agent that has been suggested as a cause of acute respiratory tract infection in children (Schildgen et al., 2008). This virus was first identified in nasopharyngeal, serum or stool samples, and was thus suggested to play a pathogenic role in some cases of Kawasaki disease (Catalano-Pons et al., 2007). Late, this work was verified by the results from another group showing that HboV can indeed be detected in nasopharyngeal secretions of KD patients, demonstrating a coincidental or possible etiological association between HboV infection and KD pathogenesis (Santos et al., 2011). Furthermore, a significant correlation between HboV infection and KD incidence was identified based on epidemiological data (Kim et al., 2014; Lim et al., 2021), whereas some investigators have proposed that there is little correlation between HboV infection and KD based on the serological test (Lehmann et al., 2009). Cytomegalovirus was also suggested to be involved in the development of atypical KD and coronary aneurysms (Catalano-Pons et al., 2005; Guc et al., 2008). Taken together, more intense researches is needed to elucidate the precise mechanism concerning DNA viruses associated with KD pathogenesis.

#### 2.1.2 RNA viruses associated with KD

Apart from the DNA viruses mentioned above, a total of nine types of RNA viruses have been suggested to correlate with KD pathogenesis, including coxsackie virus, enterovirus, human coronavirus NL63 (HCoV-NL63), influenza virus, measles virus, SARS-CoV-2 (severe acute respiratory syndrome coronavirus-2), feline virus, influenza A virus H1N1 and human immunodeficiency virus, as discussed below.

##### 2.1.2.1 Coxsackie virus

The coxsackie virus, which belongs to enteroviruses of small RNA viridine, has been identified as the main cause of viral myocarditis in humans since 1955 (Dalldorf, 1955). Both coxsackie virus B3 (CVB3) and coxsackie virus A4 were identified to correlate with KD (Rigante et al., 2012; Ueda et al., 2015), and this type of virus can induce neonatal symptoms similar to viral myocarditis observed in KD (Verma et al., 2009).

##### 2.1.2.2 Enterovirus

It has been demonstrated that the KD incidence in the enterovirus (EV)-infected cohort was significantly higher than that in the non-EV-infected cohort (Weng et al., 2018), thereby indicating a high correlation between EV infection and KD. In addition, a decreased incidence of severe enterovirus infection cases is simultaneously correlated with decreased KD hospitalizations during the SARS-CoV-2 epidemic (Guo et al., 2022), thus suggesting that enterovirus might function as a critical mediator during the pathogenesis of KD.

##### 2.1.2.3 HCoV-NL63 virus

Although HCoV-NL63 was once identified in several KD patients (Dominguez et al., 2006), most data later do not support an association between HCoV-NL63 infection and KD (Baker et al., 2006; Chang et al., 2006; Lehmann et al., 2009). In fact, only 1 (2%) of 48 patients with KD was found to be positive for HCoV-NL63/NH (Shimizu et al., 2005), although HCoV-229E was also suggested to be involved in KD (Lehmann et al., 2009; Shirato et al., 2014).

##### 2.1.2.4 Influenza virus

Influenza viruses have been revealed to positively correlate with the monthly KD incidence (Kim et al., 2014). For instance, influenza A H1N1/09 virus has been shown to be associated with the pathogenesis of KD by several groups (Joshi et al., 2011; Wang et al., 2019; Banday et al., 2021). Additionally, Parainfluenza type 3 virus (PIV-3) was also found to correlate with KD (Schnaar and Bell, 1982; Karron et al., 1993), suggesting that influenza virus infection has etiological importance in the development of KD. However, given that concomitant influenza infection affects the clinical manifestations of KD and impacts the laboratory test results of the disease (Huang et al., 2015), it remains to be determined regarding influenza infection and KD pathogenesis.

##### 2.1.2.5 Measles virus

The measles virus (MeV), which is an enveloped RNA virus, frequently causes acute febrile illness accompanied by a rash (Takemoto et al., 2022). This virus can be isolated from KD children, and the symptoms caused by MeV infection partially resemble those observed in KD patients (Whitby et al., 1991; Kuijpers et al., 2000).

##### 2.1.2.6 SARS-CoV-2

The RNA respiratory virus SARS-CoV-2 (severe acute respiratory syndrome coronavirus 2) can induce multisystem inflammatory syndrome in children (also called MIS-C), including multifocal endovascular dermatitis, thrombosis, and systemic thrombotic microangiopathy, which resemble certain features observed in KD (Ackermann et al., 2020; Consiglio et al., 2020; Loomba et al., 2020; Bukulmez, 2021; Cherqaoui et al., 2021; Sancho-Shimizu et al., 2021; Sokolovsky et al., 2021; Zhang et al., 2021).

Moreover, SARS-CoV-2 can be detected in certain KD patients, and a host of SARS-CoV-2-positive patients exhibit KD-like syndrome (Consiglio et al., 2020; Jones et al., 2020; Toubiana et al., 2020; Sharma et al., 2021). However, although high titers of anti-SARS-CoV-2 antibodies have been detected both in KD and multisystem inflammatory syndrome patients (Kabeerdoss et al., 2021), the two diseases are different because of the differential T-cell subsets, interleukin (IL)-17A, and biomarkers associated with arterial damage (Consiglio et al., 2020). On the other hand, global studies have reported that the incidence of KD declined during the COVID-19 pandemic, suggesting a potential KD pathogenesis involving transmission among children (Ae et al., 2022). However, several earlier studies showed that the KD incidence has increased during the pandemic (Ouldali et al., 2020; Roe, 2020; Stower, 2020; Viner and Whittaker, 2020), supporting the hypothesis that KD might be caused by an unknown RNA virus that may function as the main trigger in inducing abnormal immune responses in genetically susceptible individuals.

##### 2.1.2.7 Other types of RNA viruses

In addition to the RNA viruses mentioned above, several other types of RNA viruses were also found to be involved in KD. For example, both a novel feline virus (Moynahan, 1987) and the influenza A virus (Wang et al., 2019) were suggested to be related to KD symptoms. Notably, HIV patients also show symptoms similar to those observed in KD patients (Johnson et al., 2016). The intracytoplasmic inclusion bodies induced by viruses can be isolated from KD patients, suggesting that the infectious etiologic agent of KD might be associated with an unknown novel RNA virus (Rowley et al., 2011). In addition, dengue virus was also identified in the serum of certain KD patients in southern Thailand, and mosquitoes were hypothesized to work with the dengue virus to spread the KD pathogen, thus inducing cell proliferation and morphological changes in endothelial cells and coronary arteritis lesions in KD patients (Sopontammarak et al., 2008). Moreover, regions with the highest reported arboviral infections in Venezuela simultaneously have the highest incidence of KD (Paniz-Mondolfi et al., 2020), demonstrating the critical roles of viral infections in mediating the pathogenesis of KD.

### 2.2 Bacterial infection associated with KD

Regarding bacterial infection, the superantigens produced by gut bacteria may be involved in the onset of KD. Until recently, there were five *Streptococcus* spp. (*S. pneumonia*, *pseudopneumoniae, oralis, gordonii, and sanguinis*) were found to increase during the acute phase in KD patients based on metagenomic sequencing, indicating that Streptococci are involved in the pathogenesis of KD disease (Kinumaki et al., 2015). Furthermore, the stool of KD patients contains higher numbers of gram-positive bacteria, including *Streptococcus*, *Staphylococcus*, *Eubacterium*, and *Peptostreptococcus* genera, Hsp60-producing gram-negative bacteria, and a lower number of lactobacilli, when compared with those from healthy control children (Yamashiro et al., 1996; Takeshita et al., 2002; Nagata et al., 2009). Specifically, three pathogens, *S. pyogenes* (Leahy et al., 2012), *S. mitis* Nm-65 (Tabata et al., 2021), and *S. sanguis* (Tsurumizu et al., 1991), have been identified in the pleural fluid, tooth surface or blood of KD patients. Additionally, serum IgM antibodies against superantigens of *S. aureus* and *S. pyogenes* have been identified in KD patients (Matsubara et al., 2006), and these two pathogens together can produce 19 different superantigens (Llewelyn and Cohen, 2002). Mechanistically, *S. aureus* isolated from the rectum or pharynx of KD patients can secrete toxic shock syndrome toxin 1 (TSST-1) and staphylococcal protein A, which in turn stimulate Vβ2^+^ lymphocyte amplification and are thus involved in the abnormal immune responses of KD patients (Leung et al., 1993; Wann et al., 1999; Leung et al., 2002).

Regarding *Yersinia pseudotuberculosis* (Konishi et al., 1997), the *Propionibacterium acnes* strain and its products cytopathogenic proteins (CPPs; Kato et al., 1983; Tomita et al., 1987) can all be isolated from KD patients, suggesting a causative role of bacterial infection in mediating the pathogenesis of KD. Moreover, several recent studies suggest that *Y. pstb* infection is closely related to KD pathogenesis (Kato et al., 2019; Kamura et al., 2020; Miyata et al., 2022; Ohnishi et al., 2022), and the antibody titers of *Y. pstb* were significantly elevated in both Chinese and Japanese KD patients (Chou et al., 2005; Tahara et al., 2006). In contrast, a recent study showed that the positive rate of *Y. pstb* infection is much lower in KD patients (Horinouchi et al., 2015; Hayashi et al., 2023), but when the population is exposed to a higher risk of *Y. pstb* infection, the incidence of KD is much higher (Vincent et al., 2007).

### 2.3 *Mycoplasma pneumoniae* and *Chlamydia pneumoniae* hypothesis related to KD

In addition to the microbes mentioned above, *M. pneumoniae* infection was identified in an important proportion of KD patients (Umezawa et al., 1989; Ebrahim et al., 2011; Lee et al., 2011; Tang et al., 2016; Wang et al., 2021; Huang et al., 2022). For instance, the *M. pneumoniae* infection-positive rate in KD patients was significantly higher than that in non-KD patients during the SARS-CoV-2 epidemic (Ding et al., 2021), and certain KD patients were found to be coinfected with *M. pneumoniae* and Epstein–Barr virus (Huang et al., 2012).

Additionally, the positive rate of serum *Chlamydia pneumoniae* IgM antibody in KD children was significantly higher than that in the control group (Numazaki and Chiba, 1996); however, another study showed that the link between *C. pneumoniae* infection and KD pathogenesis or coronary artery lesions remains to be clarified (Chua et al., 2000; Strigl et al., 2000), suggesting that more intense research is needed to confirm the correlations between *M. pneumoniae* or *C. pneumoniae* infection and KD pathogenesis.

### 2.4 Rickettsia infection and KD

Rickettsia-like organisms were also found in biopsies of the skin and lymph nodes of KD patients (Tasaka and Hamashima, 1978). However, in most cases, only *Coxiella burnetiid* but not *Rickettsia conorii*, *R. typhi*, *Coxiella burnetii* or *Ehrlichia phagocytophila* was suggested to cause KD-like symptoms in young children (Kafetzis et al., 2001), suggesting its specific causative roles in KD pathogenesis.

### 2.5 Pathogen infection evidenced from experimental studies with a murine model

Given that the fungus *Candida albicans* can be isolated from KD patients, and its extract, the *Candida albicans* water soluble fraction (CAWS) intraperitoneally injected in mice could induce symptoms resembling those observed in KD patients (Murata, 1979; Martinez et al., 2012; Yoshikane et al., 2015; Stock et al., 2016; Noval Rivas and Arditi, 2020). Furthermore, β-glucan, which is the major component of CAWS, is also increased in KD patients (Ishibashi et al., 2014). Mechanistically, the mannoprotein-β-glucan complex of *C. albicans* can affect the functions of leukocytes, endothelial cells, and platelets *in vitro* (Kurihara et al., 2003). The systematic vasculitis induced by CAWS in mice can be alleviated after administration of human immunoglobulin or etanercept (Takahashi et al., 2010b; Ohashi et al., 2013). Together, these findings imply that infectious agents might play critical roles in triggering this disease.

Another major KD-like murine coronary arteritis model involves induction by *L. casei* cell wall extract (LCWE), which is widely used to mimic systematic vasculitis in KD patients (Lehman et al., 1988; Abe et al., 2020). In the LCWE-induced mouse model, the TLR2 and ILβ-dependent signaling pathways were suggested to play important roles during its pathogenesis (Rosenkranz et al., 2005; Lee et al., 2012; Matundan et al., 2019). Additionally, the dectin-1/Syk signaling pathway in macrophages (Lin et al., 2013) and the Notch4 signaling pathway in endothelial progenitor cells are also involved in LCWE-induced coronary artery disease, thereby contributing to the development of KD pathogenesis (Wang et al., 2016). Moreover, LCWE was likewise suggested to function as immunogenic for proinflammatory T helper (Th) 1, Th17, and CD8^+^ T cells and inducible regulatory T cells (iTreg) (Noval Rivas et al., 2017; Hsieh et al., 2021). Taken together, the systematic vasculitis induced by CAWS or LCWE in mice resembles pathological features observed in KD patients, demonstrating the causative roles of etiological agent infection and related PAMP/MAMP signaling activation in inducing KD vasculitis (Table 1).

**Table 1:** Microbial etiology demonstrated as critical triggers of Kawasaki disease.

Pathogens	Related to KD	References
**Virus**		
*Epstein–barr Virus* (EBV)	EBV infection is associated with recurrence of KD	Kikuta et al. (1990)
	EBV infection is associated with the development of coronary aneurysms in KD	Kikuta et al. (1993)
	EBV infection was first demonstrated in KD cases by PCR	Rosenfeld et al. (2020)
*Adenovirus*	The cause of KD was not proved to be adenovirus by TaqMan PCR test	Shike et al. (2005)
	The specific immune response to HADV-3 plays a key role in the occurrence of KD	Fukuda et al. (2017)
*Human parvovirus B19* (HPV-B19)	HPV-B19 can cause some symptoms resembles to those observed in KD	Nigro et al. (1994)
	Infection with HPV-B19 is closely associated with KD and collagen diseases	Holm et al. (1995) and Oura et al. (2022)
	HPV-B19 DNA was identified in the blood and pathological tissues of adult KD patients	Flossdorf et al. (2020)
*Torque Teno virus 7*	A low copy number torque teno virus 7 was detected in cervical lymph nodes of a KD case by using real-time PCR	Katano et al. (2012)
	TTV7 variants were detected by metagenomic sequencing and PCR method in two KD patients	Thissen et al. (2018)
*Herpes virus*	Patients with KD and HHV6 infection had similar skin changes at the BCG vaccination site	Kakisaka et al. (2012)
	The number of KD patients increased significantly due to HHV-6 infection during the SARS-CoV-2 epidemic	Dursun and Temiz (2020)
	A child with incomplete KD complicated with HHV-6B infection developed aseptic meningitis	Alramadhan et al. (2020)
*Varicella Zoster Virus*	A case of KD patient was found to infect with EB virus and varicella-zoster virus	Turkay et al. (2006)
	A case of KD patient was found to complicate with varicella-zoster virus infection	Toprak et al. (2015)
*Human boca virus (HboV)*	Certain KD patients were found to infect with HboV by using PCR method	Catalano-Pons et al. (2007)
	The serological data shows no association between HBoV infection and KD occurrence	Lehmann et al. (2009)
	Human boca virus DNA was identified in the nasopharyngeal secretions of a male child with KD	Santos et al. (2011)
	KD was significantly correlated with the monthly incidence of human boca virus	Kim et al. (2014)
*Cytomegalovirus*	Two infants with cytomegalovirus infection developed atypical KD and coronary aneurysm	Catalano-Pons et al. (2005)
	A case of atypical KD was found to infect with cytomegalovirus	Guc et al. (2008)
*Dengue virus*	The dengue virus titer is positive in certain KD children	Sopontammarak et al. (2008)
*Coxsackie virus*	The *coxsackie virus* infection was found in two cases of KD by using ELISA method, and the CVB3 antibody was detected by complement binding assay	Rigante et al. (2012)
	The antibody titer to coxsackie virus A4 was significantly higher than those in an adult KD case	Ueda et al. (2015)
*Enterovirus*	The cumulative incidence of KD in enterovirus-infected cohort was significantly higher than that in non-EV-infected cohort	Weng et al. (2018)
	The decrease in the number of KD hospitalizations was positively correlated with the decrease in the number of severe enterovirus infections	Guo et al. (2022)
HCoV*-*NL63	A lack of evidence proving human coronavirus NL63 infection associated with KD induction	Shimizu et al. (2005)
	The infection rate of HCoV-NL63 in KD patients is very low	Dominguez et al. (2006)
	Lack of association between infection with HCoV*-*NL63 virus and KD	Chang et al. (2006)
	Serological data showed no association with HCoV-NL63 infection in KD children	Lehmann et al. (2009)
	Serological data support that HCoV-NL63 is not involved in KD, but suggest that HCoV-229E may be involved in KD	Shirato et al. (2014)
*Parainfluenza type3 virus*	The parainfluenza type 3 virus infection was suggested to associate with KD occurrence	Schnaar and Bell (1982)
	The parainfluenza virus type 3 infection was found to associate with KD in one patient	Johnson and Azimi (1985)
*Measles-virus*	The measles-virus infection was suggested to associate with KD occurrence	Whitby et al. (1991)
*SARS-CoV-2*	The KD incidence has increased during the SARS-CoV-2 pandemic	Ouldali et al. (2020), Sandhaus et al. (2020), and Stower (2020)
	The cases of SARS-CoV-2 infection children have a higher frequency of myocarditis or pericarditis than the classic KD	Ventura et al. (2020)
	The KD incidence is increased during the pandemic of SARS-CoV-2 or influenza A H1N1 in western counties	Kam et al. (2020)
	The SARS-CoV-2 cases show a highly active proinflammatory cytokine response similar to KD	Choi (2020)
	Asymptomatic children with SARS-CoV-2 infection shows a hyperinflammatory syndrome similar to KD shock syndrome	Rehman et al. (2020)
	Several concurrent incomplete KD cases with SARS-CoV-2 infection were identified	Rivera-Figueroa et al. (2020) and Raut et al. (2021)
*Feline virus*	KD was suggested to correlate with a new feline virus transmitted by fleas	Moynahan (1987)
*H1N1*	The H1N1 virus infection was identified in the cases of incomplete KD patients	Wang et al. (2019)
*Human immunodeficiency virus*	The inflammatory characteristics of pediatric KD resembles the symptoms of adult immunodeficiency virus syndrome	Johnson et al. (2016)
*Virus-like particles*	The virus-like particles were found in the circulating peripheral blood of KD patients	Lin et al. (1992)
	The accumulation of virus-like particles (VLP) in lung tissue of KD patients, and the intracytoplasmic inclusion bodies of skin cells in the ciliated bronchial wall of KD patients were suggested to be induced by virus-like particles	Rowley et al. (2011)
**Bacteria**		
*Staphylococcus aureus*	The amplification of T cells in KD patients may be caused by a new clone of TSST-producing *S. aureus*	Leung et al. (1993)
	High levels of extracellular SpA secreted locally by *S. aureus* in the gastrointestinal tract may lead to KD-like symptoms	Wann et al. (1999)
	The value of *S. aureus* in larynx and rectum mucosa was higher in KD patients	Abe et al. (2003)
	Staphylococcus superantigens is associated with KD pathogenesis	Matsubara and Fukaya (2007)
	Multiple superantigens are involved in KD by using serum IgG and IgM antibodies against all the superantigens	Matsubara et al. (2006)
*Yersinia pseudotuberculosis* (*Y. pstb*)	*Y. pseudotuberculosis* was isolated from the stool of a KD patient	Konishi et al. (1997)
	Some studies have shown that superantigen (YPM) is produced *in vivo* and plays an important role in the pathogenesis of pseudomonas tuberculosis infection	Abe et al. (1997)
	KD was significantly associated with myocarditis and the increase of yersinia antibody titer	Chou et al. (2005)
	The incidence of coronary artery lesions in *Y. pseudotuberculosis* positive group was significantly higher than that in Yersinia negative group in KD patients	Tahara et al. (2006)
	The KD incidence is higher when the population is exposed to the risk of *Y. pseudotuberculosis* infection	Vincent et al. (2007)
	Specific molecules in the serum samples of KD share a common molecular structure with the microbe associated molecular pattern (MAMP) of *Y. pseudotuberculosis*	Kusuda et al. (2014)
	KD Patients associated with *Y. pseudotuberculosis* infection had significantly more frequent cardiac sequelae (CS)	Horinouchi et al. (2015)
	A KD-like patient who was positive for yersinia tuberculosis was diagnosed with far east scarlet fever	Ocho et al. (2018)
	LOOP-mediated isothermal amplification method identifies *Y. pseudotuberculosis* infection in KD patient	Kato et al. (2019)
	The symptom caused by pseudomeric *mycobacterium tuberculosis* infection resembles o the features observed in KD	Kamura et al. (2020)
*Propionibacterium acnes*	The levels of anti-cytopathic protein (CPP) antibodies in serum of KD patients are increased during the acute phase	Tomita et al. (1987)
	The variant strain of *P. acnes* may have a causative role in KD and house-dust mites a role as vectors	Kato et al. (1983)
*Rickettsia-Lick organism*	The clostridium bursteni is associated with KD instead of other rickettsiae pathogen	Kafetzis et al. (2001)
*Streptococcus sanguis*	Streptococcus hemorrhage can be isolated from KD patients in acute stage	Tsurumizu et al. (1991)
*Bacillus cereus*	KD specific molecules in serum share a common molecular structure with the MAMP of *B. cereus*.	Kusuda et al. (2014)
*Lactobacillus casei*	The asymmetric inflammatory coronary inflammation was detected in the LCWE-induced mouse model	Lehman et al. (1988)
	The macrophage dectin-1/Syk-mediated pathway is involved in LCWE-induced CALs and production of IL-6 and MCP-1	Lin et al. (2013)
	CD8^+^ T cells functionally contributed to the development of KD vasculitis in LCWE-induced mouse model	Noval Rivas et al. (2017)
	The endothelial progenitor cell Notch4 signaling pathway was identified in the LCWE-induced mouse model	Wang et al. (2016)
	The adrenergic stimulation after KD vasculitis can cause myocardial hypertrophy and bridging fibrosis in the LCWE-induced mouse model	Matundan et al. (2019)
	The electrophysiological abnormalities and cardiac neuronal remodeling were observed in the LCWE-induced mouse model	Abe et al. (2020)
	The coronary artery stenosis with severe coronary vasculitis and elastin degradation was detected in the LCWE-induced mouse model	Suganuma et al. (2020)
	Nonpathogenic LCWE-specific T-cell combinations are related to KD occurrence	Hsieh et al. (2021)
*Mycobacterium SSP*	Atypical mycobacterium infection enhances autoimmunity leading to coronary arteritis after vaccination with BCG	Nakamura et al. (2007)
	The development of tuberculid in the two infants might be associated with the remnant immune activation of KD	Yamada et al. (2016)
*Streptococcus*	Group A streptococcus was not detected in an adult Japanese female with KD	Hattori et al. (2005)
	The superantigens of *S. pyogenes* are involved in KD pathogenesis based on the IgM antibodies test	Matsubara et al. (2006)
	KD is associated with many streptococcal superantigens	Matsubara and Fukaya (2007)
	The first case of incomplete KD complicated with *S. pyogenes* pneumonia was reported	Leahy et al. (2012)
	The complete genome sequence of *Streptococcus* nM-65 can be isolated from KD patient	Tabata et al. (2021)
**Fungi**		
*Candida albicans*	The fungi *C. albicans* isolated from KD patients can produce coronary arteritis in mice	Murata (1979)
	CAWS strongly inhibits leukocyte function *in vitro*	Kurihara et al. (2003)
	The adeno-associated virus vector encoding IL-10 improves CAWS-induced cardiac dysfunction and lethality in mouse	Nakamura et al. (2018)
	The genetic background of CAWS immune response is related to the occurrence of coronary arteritis	Nagi-Miura et al. (2004)
	The genetic control of susceptibility to induction of vasculitis by the *candida albicans* extract is dependent on the mouse strains, but is not linked to the histocompatibility-2 loci	Takahashi et al. (2004)
	Severe stenosis of the aorta and coronary arteries, and fibrinoid necrosis in the vessel walls were observed in the CAWS-induced DBA/2 mouse strain	Hirata et al. (2006)
	Most CAWS strains can induce vasculitis	Nagi-Miura et al. (2008)
	*Candida* cell wall mannan might contribute to coronary arteritis and acute shock, and that an alteration of mannan structure could be responsible for Candida pathogenicity	Tada et al. (2008)
	The human immunoglobulin suppresses development of murine systemic vasculitis induced by CAWS	Takahashi et al. (2010b)
	The important role of CCR2 involved in the pathogenesis of CAWS-induced mouse model	Martinez et al. (2012)
	Etanercept is effective in inhibiting CAWS-induced vasculitis and may be a new therapeutic drug for KD	Ohashi et al. (2013)
	The preformed toxins and the *Candida* species were identified as the dominant fungus leading to KD	Rodo et al. (2014)
	The α-mannan contained in *C. albicans* extract could induce coronary arteritis and acute shock	Tada et al. (2014)
	KD patients have a higher titer of β -glucan (BG) antibody against candida cell wall	Ishibashi et al. (2014)
	Granulocyte/macrophage colony stimulating factor was found in the CAWS-induced cardiac inflammation site of KD mice	Stock et al. (2016)
	The CAWS-induced mouse model showed inflammatory cell infiltration, destruction of elastic lamellae, loss of medial smooth muscle cells and intimal thickening, whose features resembles the vascular lesions of KD patients	Yoshikane et al. (2015)
	The recognition of A-mannan by A-mannan receptor dectin-2 plays an important role in the pathogenesis of vasculitis in KD mice induced by *C. albicans* cell wall polysaccharide.	Oharaseki et al. (2020)
	The mannoprotein fractions of clinically isolated Candida species can induce vasculitis in mice	Tanaka et al. (2020)
	The cell wall mannoprotein of *C. krusei* could induce coronary vasculitis in mouse model	Yanai et al. (2020)
**Mycoplasma**		
*Mycoplasma pneumoniae*	The pulmonary symptoms of KD were suggested to be associated with pneumococcal infection	Lee et al. (2011)
	Incomplete KD patients were found to be related with acute *M. pneumoniae* infection	Ebrahim et al. (2011)
	Many cases of KD simultaneously infected with Epstein–barr virus and *M. pneumoniae* were identified	Huang et al. (2012)
	MP infection occurs in the elderly population, and the respiratory tract involvement rate is higher in KD patients	Tang et al. (2016)
	The MP infection rate in KD patients was significantly higher than that those observed in non-KD patients	Ding et al. (2021)
	*M. pneumoniae* infection may be associated with a reduced incidence of small CAA in KD patients	Wang et al. (2021)
	The serological test for *M. pneumoniae* infection was positive in one case of acute KD patients	Huang et al. (2022)
	A lack of evidence showing association between *M. pneumoniae* infection and KD induction	Strigl et al. (2000)
**Chlamydia**		
	The positive rate of serum *C. pneumoniae* IgM antibody in KD was higher than those in control group	Numazaki and Chiba (1996)
	A deficiency evidence of *C. pneumoniae* infection associated with KD occurrence	Chua et al. (2000)

## 3 Summary and perspective

**Figure 1:**
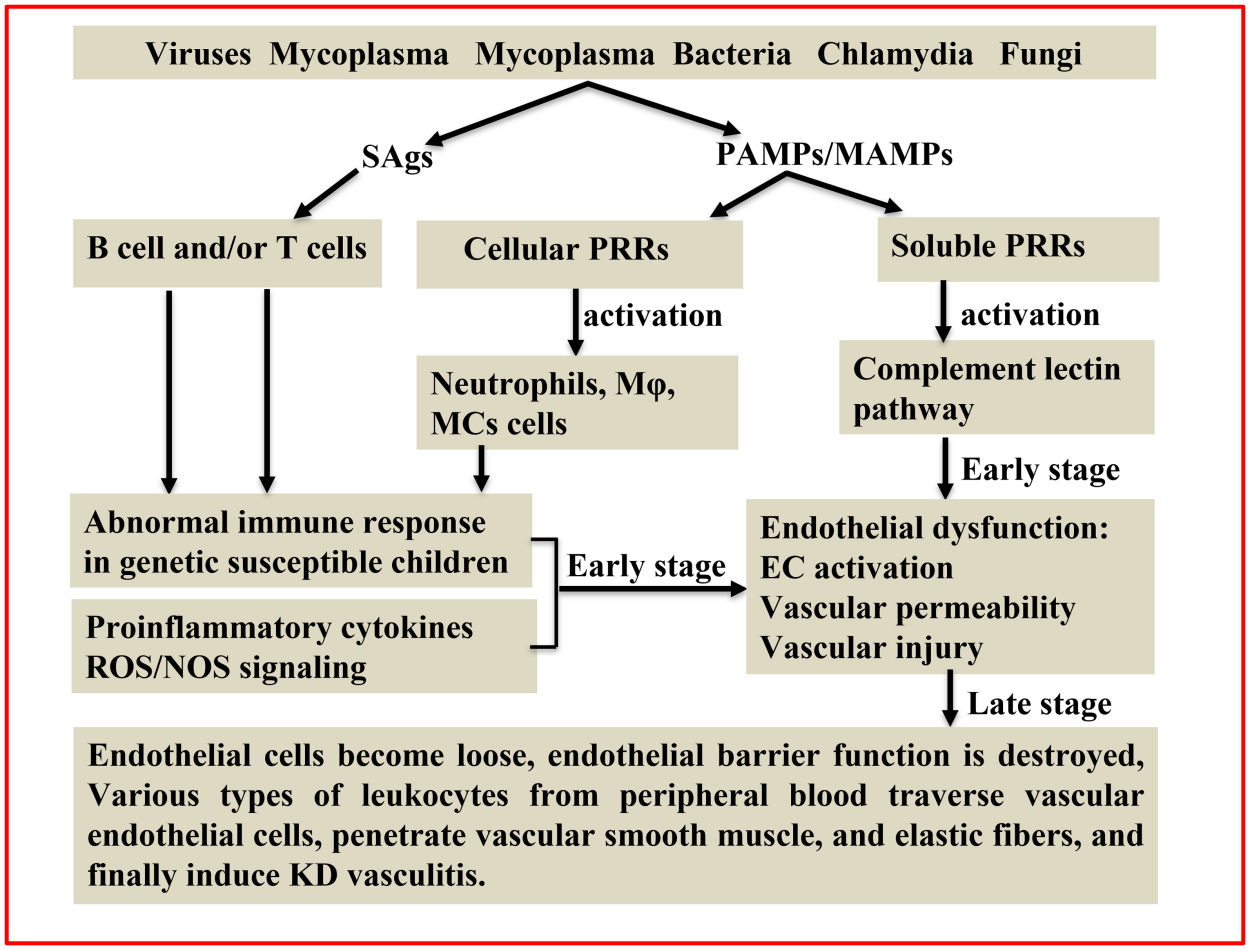
Schematic illustrating the pathogenic mechanisms of KD. The superantigens (SAgs) hypothesis and different infectious agents produce pathogen/microbe-associated molecular patterns (PAMPs/MAMPs) were all proposed to be involved in KD pathogenesis. SAgs non-specifically activate T cells and/or B cells. PAMPs/MAMPs also stimulate immune cells [e.g., macrophages (Mφ), dendritic cells (DCs), monocytes (MCs)] and endothelial cells (ECs) through cellular pattern recognition receptors (PRRs; e.g., TLRs, NOD1, and Dectin-1/-2). Additionally, PAMPs/MAMPs can activate the complement lectin pathway through soluble PRRs (e.g., ficolin-1 and mannose binding lectin-2). Activated complement pathways can induce inflammatory vascular damage through recruitment of innate inflammatory cells and direct injury to ECs. This cross-talk among different cells augments the production of proinflammatory cytokines/chemokines and reactive oxygen/nitrogen species (ROS/NOS), hence leading to a systemic inflammatory reaction in KD.

Taken together, various pathogens identified in KD were all suggested to be the critical triggers in causing systematic vasculitis, and these pathogens were demonstrated to work independently or synergistically to potentiate abnormal immune responses by inducing pyroptosis and/or proinflammatory cell death, hence leading to systematic vasculitis in KD (Figure 1). However, whether these pathogens are direct causes or merely the accompanying pathogens after KD induction remains elusive. Additionally, the causative agent of KD remains ambiguous, and several questions remain to be clarified. First, those pathogens suggested to be involved in the pathogenesis of KD largely rely on PCR and serological methods using a relatively small sample size. Second, the differences in timing of obtaining the blood sample and constraints of the study design used to measure pathogens in KD patients by different investigators could make pathogen identification inconsistent. Third, whether KD is caused by a single pathogen or is the combined result of more than one agent remains to be investigated. Consequently, the relationship between pathogen infection and KD vasculitis is far more complex than currently appreciated. Caution should be exercised in the clinic when considering the possible agents merely based on the symptom similarities between KD and other infectious diseases. Importantly, given that the recognition of the infectious origin of KD is a critical prerequisite to understanding its pathogenetic mechanism, more intense research using artificial intelligence, metagenomic sequencing and culturing specific pathogens isolated from KD patients from multiple centers and then verifying each of them in animal models could help uncover the underlying mechanisms of pathogen infections involved and thus facilitate the development of novel intervention strategies for Kawasaki disease.

## Author contributions

WW: Writing – original draft, Data curation. LZ: Conceptualization, Funding acquisition, Investigation, Writing – original draft. XL: Writing – original draft, Data curation, Project administration. ZL: Data curation, Resources, Writing – original draft. HL: Conceptualization, Investigation, Supervision, Writing – review & editing. GQ: Conceptualization, Funding acquisition, Writing – review & editing.

## References

[ref1] Abe J. Onimaru M. Matsumoto S. Noma S. Baba K. Ito Y.. (1997). Clinical role for a superantigen in *Yersinia pseudotuberculosis* infection. J. Clin. Investig. 99, 1823–1830. doi: 10.1172/jci119349, PMID: 9109426 PMC508006

[ref2] Abe M. Rastelli D. D. Gomez A. C. Cingolani E. Lee Y. Soni P. R.. (2020). IL-1-dependent electrophysiological changes and cardiac neural remodeling in a mouse model of Kawasaki disease vasculitis. Clin. Exp. Immunol. 199, 303–313. doi: 10.1111/cei.13401, PMID: 31758701 PMC7008220

[ref3] Abe J. Terai M. Nogami H. Toyoda Y. Nakajima H. Nakano T.. (2003). Colonization of the superantigen-producing *Staphylococcus aureus* among patients with Kawasaki disease. Pediatr. Res. 53:168. doi: 10.1203/00006450-200301000-00088

[ref4] Ackermann M. Verleden S. E. Kuehnel M. Haverich A. Welte T. Laenger F.. (2020). Pulmonary vascular Endothelialitis, thrombosis, and angiogenesis in Covid-19. N. Engl. J. Med. 383, 120–128. doi: 10.1056/NEJMoa2015432 32437596 PMC7412750

[ref5] Ae R. Makino N. Kuwabara M. Matsubara Y. Kosami K. Sasahara T.. (2022). Incidence of Kawasaki disease before and after the COVID-19 pandemic in Japan: results of the 26th Nationwide survey, 2019 to 2020. JAMA Pediatr. 176, 1217–1224. doi: 10.1001/jamapediatrics.2022.3756, PMID: 36251290 PMC9577881

[ref6] Alramadhan M. M. Kamdar A. A. Lafferty-Prather M. Aguilera E. A. Wootton S. H. (2020). Incomplete Kawasaki disease associated with human herpes Virus-6 variant B infection and aseptic meningitis. Glob Pediatr Health 7:2333794X20939759. doi: 10.1177/2333794X20939759, PMID: 32782920 PMC7383600

[ref7] Ariza M. E. Glaser R. Kaumaya P. T. Jones C. Williams M. V. (2009). The EBV-encoded dUTPase activates NF-kappa B through the TLR2 and MyD88-dependent signaling pathway. J. Immunol. 182, 851–859. doi: 10.4049/jimmunol.182.2.851, PMID: 19124728 PMC12892303

[ref8] Baker S. C. Shimizu C. Shike H. Garcia F. van der Hoek L. Kuijper T. W.. (2006). Human coronavirus-NL63 infection is not associated with acute Kawasaki disease. Adv. Exp. Med. Biol. 581, 523–526. doi: 10.1007/978-0-387-33012-9_94, PMID: 17037590 PMC2868826

[ref9] Baldari C. T. Onnis A. Andreano E. Del Giudice G. Rappuoli R. (2023). Emerging roles of SARS-CoV-2 spike-ACE2 in immune evasion and pathogenesis. Trends Immunol. 44, 424–434. doi: 10.1016/j.it.2023.04.001, PMID: 37137805 PMC10076505

[ref10] Banday A. Z. Arul A. Vignesh P. Singh M. P. Goyal K. Singh S. (2021). Kawasaki disease and influenza-new lessons from old associations. Clin. Rheumatol. 40, 2991–2999. doi: 10.1007/s10067-020-05534-1, PMID: 33387094 PMC7778392

[ref11] Bukulmez H. (2021). Current understanding of multisystem inflammatory syndrome (MIS-C) following COVID-19 and its distinction from Kawasaki disease. Curr. Rheumatol. Rep. 23:58. doi: 10.1007/s11926-021-01028-4, PMID: 34216296 PMC8254432

[ref12] Burns J. C. Herzog L. Fabri O. Tremoulet A. H. Rodo X. Uehara R.. (2013). Seasonality of Kawasaki disease: a global perspective. PloS One 8:e74529. doi: 10.1371/journal.pone.0074529, PMID: 24058585 PMC3776809

[ref13] Catalano-Pons C. Giraud C. Rozenberg F. Meritet J. F. Lebon P. Gendrel D. (2007). Detection of human bocavirus in children with Kawasaki disease. Clin. Microbiol. Infect. 13, 1220–1222. doi: 10.1111/j.1469-0691.2007.01827.x, PMID: 17850342

[ref14] Catalano-Pons C. Quartier P. Leruez-Ville M. Kaguelidou F. Gendrel D. Lenoir G.. (2005). Primary cytomegalovirus infection, atypical Kawasaki disease, and coronary aneurysms in 2 infants. Clin. Infect. Dis. 41, E53–E56. doi: 10.1086/432578, PMID: 16080076

[ref15] Chang L. Y. Chiang B. L. Kao C. L. Wu M. H. Chen P. J. Berkhout B.. (2006). Lack of association between infection with a novel human coronavirus (HCoV), HCoV-NH, and Kawasaki disease in Taiwan. J Infect Dis 193, 283–286. doi: 10.1086/498875, PMID: 16362893 PMC7109937

[ref16] Chang L.-Y. Lu C.-Y. Shao P.-L. Lee P.-I. Lin M.-T. Fan T.-Y.. (2014). Viral infections associated with Kawasaki disease. J. Formos. Med. Assoc. 113, 148–154. doi: 10.1016/j.jfma.2013.12.008, PMID: 24495555 PMC7125523

[ref17] Chang L. S. Yan J. H. Li J. Y. Yeter D. D. Huang Y. H. Guo M. M.. (2020). Blood mercury levels in children with Kawasaki disease and disease outcome. Int. J. Environ. Res. Public Health 17:3726. doi: 10.3390/ijerph17103726, PMID: 32466179 PMC7277186

[ref18] Cherqaoui B. Kone-Paut I. Yager H. Bourgeois F. L. Piram M. (2021). Delineating phenotypes of Kawasaki disease and SARS-CoV-2-related inflammatory multisystem syndrome: a French study and literature review. Rheumatology (Oxford) 60, 4530–4537. doi: 10.1093/rheumatology/keab026, PMID: 33493353 PMC7928644

[ref19] Choi J.-W. (2020). Can we get a clue for the etiology of Kawasaki disease in the COVID-19 pandemic? Clin Experiment Pediatr 63, 335–336. doi: 10.3345/cep.2020.00955, PMID: 32683814 PMC7462826

[ref20] Chou C. T. Chang J. S. Ooi S. E. Huo A. P. Chang S. J. Chang H. N.. (2005). Serum anti-Yersinia antibody in Chinese patients with Kawasaki disease. Arch. Med. Res. 36, 14–18. doi: 10.1016/j.arcmed.2004.09.004 15777989

[ref21] Chua P. K. Nerurkar V. R. Yu Q. G. Woodward C. L. Melish M. E. Yanagihara R. (2000). Lack of association between Kawasaki syndrome and infection with parvovirus B19, human herpesvirus 8, TT virus, GB virus C/hepatitis G virus or *Chlamydia pneumoniae*. Pediatr. Infect. Dis. J. 19, 477–479. doi: 10.1097/00006454-200005000-00019 10819350

[ref22] Consiglio C. R. Cotugno N. Sardh F. Pou C. Amodio D. Rodriguez L.. (2020). The immunology of multisystem inflammatory syndrome in children with COVID-19. Cells 183, 968–981.e7. doi: 10.1016/j.cell.2020.09.016 PMC7474869 32966765

[ref23] Corinaldesi E. Pavan V. Andreozzi L. Fabi M. Selvini A. Frabboni I.. (2020). Environmental factors and Kawasaki disease onset in Emilia-Romagna, Italy. Int. J. Environ. Res. Public Health 17:1529. doi: 10.3390/ijerph17051529, PMID: 32120916 PMC7084934

[ref24] Dalldorf G. (1955). COXSACKIE VIRUSES. Annu. Rev. Microbiol. 9, 277–296. doi: 10.1146/annurev.mi.09.100155.001425 13259467

[ref25] Ding Y.-Y. Ren Y. Qin J. Qian G.-H. Tang Y.-J. Chen Y.. (2021). Clinical characteristics of Kawasaki disease and concurrent pathogens during isolation in COVID-19 pandemic. World J. Pediatr. 17, 263–271. doi: 10.1007/s12519-021-00431-2 34160770 PMC8219783

[ref26] Dominguez S. R. Anderson M. S. Glode M. P. Robinson C. C. Holmes K. V. (2006). Blinded case-control study of the relationship between human coronavirus NL63 and Kawasaki syndrome. J Infect Dis 194, 1697–1701. doi: 10.1086/509509, PMID: 17109341 PMC7199878

[ref27] Dursun R. Temiz S. A. (2020). The clinics of HHV-6 infection in COVID-19 pandemic: Pityriasis rosea and Kawasaki disease. Dermatol. Ther. 33:e13730. doi: 10.1111/dth.13730, PMID: 32475003 PMC7300497

[ref28] Ebrahim M. Gabay M. Rivas-Chacon R. F. (2011). Evidence of acute Mycoplasma infection in a patient with incomplete and atypical Kawasaki disease: a case report. Case Rep. Med. 2011:606920. doi: 10.1155/2011/606920, PMID: 22203852 PMC3235783

[ref29] Embil J. A. McFarlane E. S. Murphy D. M. Krause V. W. Stewart H. B. (1985). Adenovirus type 2 isolated from a patient with fatal Kawasaki disease. Can. Med. Assoc. J. 132:1400. PMID: 4005729 PMC1346107

[ref30] Flossdorf S. Schiwy-Bochat K. H. Teifel D. Fries J. W. U. Rothschild M. A. (2020). Sudden death of a young adult with coronary artery vasculitis, coronary aneurysms, parvovirus B19 infection and Kawasaki disease. Forensic Sci. Med. Pathol. 16, 498–503. doi: 10.1007/s12024-020-00263-y, PMID: 32495258

[ref31] Fukuda S. Ito S. Fujiwara M. Abe J. Hanaoka N. Fujimoto T.. (2017). Simultaneous development of Kawasaki disease following acute human adenovirus infection in monozygotic twins: a case report. Pediatr. Rheumatol. Online J. 15:39. doi: 10.1186/s12969-017-0169-x, PMID: 28511718 PMC5432973

[ref32] Ghosh P. Katkar G. D. Shimizu C. Kim J. Khandelwal S. Tremoulet A. H.. (2022). An artificial intelligence-guided signature reveals the shared host immune response in MIS-C and Kawasaki disease. Nat. Commun. 13:2687. doi: 10.1038/s41467-022-30357-w, PMID: 35577777 PMC9110726

[ref33] Guc B. U. Cengiz N. Yildirim S. V. Uslu Y. (2008). Cytomegalovirus infection in a patient with atypical Kawasaki disease. Rheumatol. Int. 28, 387–389. doi: 10.1007/s00296-007-0440-4, PMID: 17717671 PMC7079931

[ref34] Guo M. M.-H. Yang K. D. Liu S.-F. Kuo H.-C. (2022). Number of Kawasaki disease admissions is associated with number of domestic COVID-19 and severe enterovirus case numbers in Taiwan. Children 9:149. doi: 10.3390/children9020149, PMID: 35204870 PMC8870605

[ref35] Hara T. Yamamura K. Sakai Y. (2021). The up-to-date pathophysiology of Kawasaki disease. Clin Transl Immunol 10:e1284. doi: 10.1002/cti2.1284, PMID: 33981434 PMC8109476

[ref36] Hattori T. Matsukawa Y. Takei M. Yamaguchi K. Yamazaki T. Sawada U.. (2005). Adult Kawasaki disease unrelated to Epstein-Barr virus and group a Streptococcus. Intern. Med. 44, 1182–1184. doi: 10.2169/internalmedicine.44.1182, PMID: 16357458

[ref37] Hayashi H. Uda K. Araki Y. Akahoshi S. Tanaka M. Miyata K.. (2023). Association of Yersinia Infection with Kawasaki Disease: a prospective multicenter cohort study. Pediatr. Infect. Dis. J. 42, 1041–1044. doi: 10.1097/INF.0000000000004084, PMID: 37725804

[ref38] Hirata N. Ishibashi K.-I. Ohta S. Hata S. Shinohara H. Kitamura M.. (2006). Histopathological examination and analysis of mortality in DBA/2 mouse vasculitis induced with CAWS, a water-soluble extracellular polysaccharide fraction obtained from *Candida albicans*. Yakugaku Zasshi 126, 643–650. doi: 10.1248/yakushi.126.643, PMID: 16880722

[ref39] Holm J. M. Hansen L. K. Oxhoj H. (1995). Kawasaki disease associated with parvovirus B19 infection. Eur. J. Pediatr. 154, 633–634. doi: 10.1007/BF02079066 7588963

[ref40] Horinouchi T. Nozu K. Hamahira K. Inaguma Y. Abe J. Nakajima H.. (2015). *Yersinia pseudotuberculosis* infection in Kawasaki disease and its clinical characteristics. BMC Pediatr. 15:177. doi: 10.1186/s12887-015-0497-2, PMID: 26561332 PMC4642785

[ref41] Hsieh L.-E. Tremoulet A. H. Burns J. C. Noval Rivas M. Arditi M. Franco A. (2021). Characterization of the T cell response to *Lactobacillus casei* Cell Wall extract in children with Kawasaki disease and its potential role in vascular inflammation. Front. Pediatr. 9:633244. doi: 10.3389/fped.2021.633244, PMID: 33681107 PMC7933244

[ref42] Huang F.-L. Chang T.-K. Jan S.-L. Tsai C.-R. Wang L.-C. Lai M.-C.. (2012). Co-morbidity of Kawasaki disease. Indian J. Pediatr. 79, 815–817. doi: 10.1007/s12098-011-0589-4 22057395

[ref43] Huang S. H. Chen C. Y. Weng K. P. Chien K. J. Hung Y. M. Hsieh K. S.. (2020). Adenovirus infection and subsequent risk of Kawasaki disease: a population-based cohort study. J. Chin. Med. Assoc. 83, 302–306. doi: 10.1097/JCMA.0000000000000266, PMID: 31990817 PMC13048015

[ref44] Huang X. Huang P. Zhang L. Xie X. Xia S. Gong F.. (2015). Influenza infection and Kawasaki disease. Rev. Soc. Bras. Med. Trop. 48, 243–248. doi: 10.1590/0037-8682-0091-2015 26108000

[ref45] Huang S.-W. Lin S.-C. Chen S.-Y. Hsieh K.-S. (2022). Kawasaki disease with combined Cholestatic hepatitis and *Mycoplasma pneumoniae* infection: a case report and literature review. Front. Pediatr. 9:8215. doi: 10.3389/fped.2021.738215, PMID: 35223706 PMC8864216

[ref46] Ishibashi K. Fukazawa R. Miura N. N. Adachi Y. Ogawa S. Ohno N. (2014). Diagnostic potential of antibody titres against Candida cell wall beta-glucan in Kawasaki disease. Clin. Exp. Immunol. 177, 161–167. doi: 10.1111/cei.12328, PMID: 24635107 PMC4089165

[ref47] Jackson H. Menikou S. Hamilton S. McArdle A. Shimizu C. Galassini R.. (2021). Kawasaki disease patient stratification and pathway analysis based on host transcriptomic and proteomic profiles. Int. J. Mol. Sci. 22:655. doi: 10.3390/ijms22115655 34073389 PMC8198135

[ref48] Johnson D. Azimi P. (1985). Kawasaki disease associated with klebsiella-pneumoniae bacteremia and Para-influenza type-3 virus-infection. Pediatr. Infect. Dis. J. 4:100. doi: 10.1097/00006454-198501000-00024, PMID: 2982131

[ref49] Johnson R. M. Bergmann K. R. Manaloor J. J. Yu X. Slaven J. E. Kharbanda A. B. (2016). Pediatric Kawasaki disease and adult human immunodeficiency virus Kawasaki-like syndrome are likely the same malady. Open Forum Infect. Dis. 3:ofw160. doi: 10.1093/ofid/ofw160, PMID: 27704015 PMC5047405

[ref50] Jones V. G. Mills M. Suarez D. Hogan C. A. Yeh D. Segal J. B.. (2020). COVID-19 and Kawasaki disease: novel virus and novel case. Hosp. Pediatr. 10, 537–540. doi: 10.1542/hpeds.2020-0123 32265235

[ref51] Joshi A. V. Jones K. D. Buckley A. M. Coren M. E. Kampmann B. (2011). Kawasaki disease coincident with influenza a H1N1/09 infection. Pediatr. Int. 53, e1–e2. doi: 10.1111/j.1442-200X.2010.03280.x, PMID: 21342333 PMC7167673

[ref52] Kabeerdoss J. Pilania R. K. Karkhele R. Kumar T. S. Danda D. Singh S. (2021). Severe COVID-19, multisystem inflammatory syndrome in children, and Kawasaki disease: immunological mechanisms, clinical manifestations and management. Rheumatol. Int. 41, 19–32. doi: 10.1007/s00296-020-04749-4, PMID: 33219837 PMC7680080

[ref53] Kafetzis D. A. Maltezou H. C. Constantopoulou I. Antonaki G. Liapi G. Mathioudakis I. (2001). Lack of association between Kawasaki syndrome and infection with *Rickettsia conorii*, *Rickettsia typhi*, *Coxiella burnetii* or *Ehrlichia phagocytophila* group. Pediatr. Infect. Dis. J. 20, 703–706. doi: 10.1097/00006454-200107000-00012 11465844

[ref54] Kakisaka Y. Ohara T. Katayama S. Suzuki T. Sasai S. Hino-Fukuyo N.. (2012). Human herpes virus type 6 can cause skin lesions at the BCG inoculation site similar to Kawasaki disease. Tohoku J. Exp. Med. 228, 351–353. doi: 10.1620/tjem.228.351, PMID: 23138414

[ref55] Kam K.-Q. Ong J. S. M. Lee J. H. (2020). Kawasaki disease in the COVID-19 era: a distinct clinical phenotype? Lancet Child Adolesc Health 4, 642–643. doi: 10.1016/s2352-4642(20)30207-8, PMID: 32622377 PMC7833489

[ref56] Kamura T. Tanaka Y. Tsumura N. Ohya T. Okamatsu Y. (2020). *Yersinia pseudotuberculosis* infection complicated with bacteremia in a 10-month-old boy. Case Rep Pediatr 2020:8846511. doi: 10.1155/2020/8846511, PMID: 33354376 PMC7737469

[ref57] Karron R. A. O'Brien K. L. Froehlich J. L. Brown V. A. (1993). Molecular epidemiology of a parainfluenza type 3 virus outbreak on a pediatric ward. J Infect Dis 167, 1441–1445. doi: 10.1093/infdis/167.6.1441, PMID: 8388907

[ref58] Katano H. Sato S. Sekizuka T. Kinumaki A. Fukumoto H. Sato Y.. (2012). Pathogenic characterization of a cervical lymph node derived from a patient with Kawasaki disease. Int. J. Clin. Exp. Pathol. 5, 814–823. PMID: 23071864 PMC3466979

[ref59] Kato H. Fujimoto T. Inoue O. Kondo M. Koga Y. Yamamoto S.. (1983). Variant strain of *Propionibacterium acnes*: a clue to the aetiology of Kawasaki disease. Lancet 2, 1383–1388. doi: 10.1016/s0140-6736(83)90921-2, PMID: 6140493

[ref60] Kato A. Miyata I. Tanaka Y. Oishi T. Teranishi H. Akaike H.. (2019). LAMP-based assay can rectify the diagnosis of *Yersinia pseudotuberculosis* infections otherwise missed by serology. J. Med. Microbiol. 68, 143–147. doi: 10.1099/jmm.0.000868, PMID: 30648936

[ref61] Kido S. Ae R. Kosami K. Matsubara Y. Makino N. Sasahara T.. (2019). Seasonality of i.v. immunoglobulin responsiveness in Kawasaki disease. Pediatr. Int. 61, 539–543. doi: 10.1111/ped.13863, PMID: 30980447

[ref62] Kikuta H. Matsumoto S. Yanase Y. Kawasaki T. Mizuno F. Osato T. (1990). Recurrence of Kawasaki-disease and epstein-barr-virus infection. J Infect Dis 162:1215. doi: 10.1093/infdis/162.5.1215, PMID: 2172398

[ref63] Kikuta H. Sakiyama Y. Matsumoto S. Hamada I. Yazaki M. Iwaki T.. (1993). Detection of epstein-barr-virus dna in cardiac and aortic tissues from chronic, active epstein-barr-virus infection associated with Kawasaki disease-like coronary-artery aneurysms. J. Pediatr. 123, 90–92. doi: 10.1016/s0022-3476(05)81546-x, PMID: 8391571

[ref64] Kikuta H. Taguchi Y. Tomizawa K. Kojima K. Kawamura N. Ishizaka A.. (1988). Epstein-Barr virus genome-positive T lymphocytes in a boy with chronic active EBV infection associated with Kawasaki-like disease. Nature 333, 455–457. doi: 10.1038/333455a0 2836734

[ref65] Kim G. B. Park S. Kwon B. S. Han J. W. Park Y. W. Hong Y. M. (2014). Evaluation of the temporal association between Kawasaki disease and viral infections in South Korea. Korean Circ J 44, 250–254. doi: 10.4070/kcj.2014.44.4.250, PMID: 25089137 PMC4117846

[ref66] Kim H. S. Shin S. W. Choi B. G. Choi H. J. (2020). Differences over 10 years in epidemiologic and clinical features of Kawasaki disease at a single tertiary center. Clin Exp Pediatr 63, 157–158. doi: 10.3345/cep.2019.01109, PMID: 32023400 PMC7170786

[ref67] Kinumaki A. Sekizuka T. Hamada H. Kato K. Yamashita A. Kuroda M. (2015). Characterization of the gut microbiota of Kawasaki disease patients by metagenomic analysis. Front. Microbiol. 6:824. doi: 10.3389/fmicb.2015.00824, PMID: 26322033 PMC4531854

[ref68] Konishi N. Baba K. Abe J. Maruko T. Waki K. Takeda N.. (1997). A case of Kawasaki disease with coronary artery aneurysms documenting *Yersinia pseudotuberculosis* infection. Acta Paediatr. 86, 661–664. doi: 10.1111/j.1651-2227.1997.tb08952.x, PMID: 9202805

[ref69] Kuijpers T. W. Herweijer T. J. Scholvinck L. Wertheim-Van Dillen P. M. Van de Veer E. M. A. (2000). Kawasaki disease associated with measles virus infection in a monozygotic twin. Pediatr. Infect. Dis. J. 19, 350–353. doi: 10.1097/00006454-200004000-00018 10783028

[ref70] Kuijpers T. W. Wiegman A. van Lier R. A. Roos M. T. Wertheim-van Dillen P. M. Pinedo S.. (1999). Kawasaki disease: a maturational defect in immune responsiveness. J Infect Dis 180, 1869–1877. doi: 10.1086/315111, PMID: 10558943

[ref71] Kuo H. C. Pan C. T. Huang Y. H. Huang F. C. Lin Y. S. Li S. C.. (2019). Global investigation of immune repertoire suggests Kawasaki disease has infectious cause. Circ. J. 83, 2070–2078. doi: 10.1253/circj.CJ-19-0206, PMID: 31378745

[ref72] Kurihara K. Shingo Y. Miura N. N. Horie S. Usui Y. Adachi Y.. (2003). Effect of CAWS, a mannoprotein-beta-glucan complex of *Candida albicans*, on leukocyte, endothelial cell, and platelet functions in vitro. Biol. Pharm. Bull. 26, 233–240. doi: 10.1248/bpb.26.233, PMID: 12576686

[ref73] Kusuda T. Nakashima Y. Murata K. Kanno S. Nishio H. Saito M.. (2014). Kawasaki disease-specific molecules in the sera are linked to microbe-associated molecular patterns in the biofilms. PloS One 9:e113054. doi: 10.1371/journal.pone.0113054, PMID: 25411968 PMC4239021

[ref74] Leahy T. R. Cohen E. Allen U. D. (2012). Incomplete Kawasaki disease associated with complicated *Streptococcus pyogenes* pneumonia: a case report. Can J Infect Dis Med Microbiol 23, 137–139. doi: 10.1155/2012/638357, PMID: 23997782 PMC3476559

[ref75] Lee M. N. Cha J. H. Ahn H. M. Yoo J. H. Kim H. S. Sohn S.. (2011). *Mycoplasma pneumoniae* infection in patients with Kawasaki disease. Korean J. Pediatr. 54, 123–127. doi: 10.3345/kjp.2011.54.3.123, PMID: 21738542 PMC3120998

[ref76] Lee Y. Schulte D. J. Shimada K. Chen S. Crother T. R. Chiba N.. (2012). Interleukin-1beta is crucial for the induction of coronary artery inflammation in a mouse model of Kawasaki disease. Circulation 125, 1542–1550. doi: 10.1161/CIRCULATIONAHA.111.072769, PMID: 22361326 PMC3337219

[ref77] Lehman T. J. A. Warren R. Gietl D. Mahnovski V. Prescott M. (1988). Variable expression of lactobacillus-casei cell wall-induced coronary arteritis—an animal-model of kawasakis disease in selected inbred mouse strains. Clin. Immunol. Immunopathol. 48, 108–118. doi: 10.1016/0090-1229(88)90161-4, PMID: 3133145

[ref78] Lehmann C. Klar R. Lindner J. Lindner P. Wolf H. Gerling S. (2009). Kawasaki disease lacks association with human coronavirus NL63 and human bocavirus. Pediatr. Infect. Dis. J. 28, 553–554. doi: 10.1097/inf.0b013e31819f41b6, PMID: 19504744

[ref79] Leung D. Y. Meissner H. C. Fulton D. R. Murray D. L. Kotzin B. L. Schlievert P. M. (1993). Toxic shock syndrome toxin-secreting *Staphylococcus aureus* in Kawasaki syndrome. Lancet 342, 1385–1388. doi: 10.1016/0140-6736(93)92752-f, PMID: 7901681

[ref80] Leung D. Y. M. Meissner H. C. Shulman S. T. Mason W. H. Gerber M. A. Glode M. P.. (2002). Prevalence of superantigen-secreting bacteria in patients with Kawasaki disease. J. Pediatr. 140, 742–746. doi: 10.1067/mpd.2002.123664, PMID: 12072880

[ref81] Li X. Chen Y. Tang Y. Ding Y. Xu Q. Sun L.. (2018). Predictors of intravenous immunoglobulin-resistant Kawasaki disease in children: a meta-analysis of 4442 cases. Eur. J. Pediatr. 177, 1279–1292. doi: 10.1007/s00431-018-3182-2, PMID: 29948255 PMC6061038

[ref82] Lim J. H. Kim Y. K. Min S. H. Kim S. W. Lee Y. H. Lee J. M. (2021). Seasonal trends of viral prevalence and incidence of Kawasaki disease: a Korea public health data analysis. J. Clin. Med. 10:3301. doi: 10.3390/jcm10153301, PMID: 34362085 PMC8347058

[ref83] Lin C. Y. Chen I. C. Cheng T. I. Liu W. T. Hwang B. Chiang B. N. (1992). VIRUS-LIKE PARTICLES WITH REVERSE-TRANSCRIPTASE ACTIVITY ASSOCIATED WITH KAWASAKI-DISEASE. J. Med. Virol. 38, 175–182. doi: 10.1002/jmv.1890380305, PMID: 1283752

[ref84] Lin M. C. Lai M. S. Jan S. L. Fu Y. C. (2015). Epidemiologic features of Kawasaki disease in acute stages in Taiwan, 1997-2010: effect of different case definitions in claims data analysis. J. Chin. Med. Assoc. 78, 121–126. doi: 10.1016/j.jcma.2014.03.009, PMID: 25636582 PMC7105041

[ref85] Lin I. C. Suen J.-L. Huang S.-K. Huang S.-C. Huang H.-C. Kuo H.-C.. (2013). Dectin-1/Syk signaling is involved in *Lactobacillus casei* cell wall extract-induced mouse model of Kawasaki disease. Immunobiology 218, 201–212. doi: 10.1016/j.imbio.2012.04.004, PMID: 22633994

[ref86] Lindquist M. E. Hicar M. D. (2019). B cells and antibodies in Kawasaki disease. Int. J. Mol. Sci. 20:1834. doi: 10.3390/ijms20081834, PMID: 31013925 PMC6514959

[ref87] Llewelyn M. Cohen J. (2002). Superantigens: microbial agents that corrupt immunity. Lancet Infect. Dis. 2, 156–162. doi: 10.1016/s1473-3099(02)00222-0, PMID: 11944185

[ref88] Loomba R. S. Villarreal E. G. Flores S. (2020). COVID-19 and Hyperinflammatory syndrome in children: Kawasaki disease with macrophage activation syndrome in disguise? Cureus 12:e9515. doi: 10.7759/cureus.9515, PMID: 32884871 PMC7462650

[ref89] Maggio M. C. Fabiano C. Corsello G. (2019). Kawasaki disease triggered by EBV virus in a child with familial Mediterranean fever. Ital. J. Pediatr. 45:129. doi: 10.1186/s13052-019-0717-8, PMID: 31627741 PMC6798734

[ref90] Martinez H. G. Quinones M. P. Jimenez F. Estrada C. Clark K. M. Suzuki K.. (2012). Important role of CCR2 in a murine model of coronary vasculitis. BMC Immunol. 13:56. doi: 10.1186/1471-2172-13-56, PMID: 23074996 PMC3519555

[ref91] Matsubara K. Fukaya T. (2007). The role of superantigens of group a Streptococcus and *Staphylococcus aureus* in Kawasaki disease. Curr. Opin. Infect. Dis. 20, 298–303. doi: 10.1097/QCO.0b013e3280964d8c, PMID: 17471041

[ref92] Matsubara K. Fukaya T. Miwa K. Shibayama N. Nigami H. Harigaya H.. (2006). Development of serum IgM antibodies against superantigens of Staphylococcus aureus and *Streptococcus pyogenes* in Kawasaki disease. Clin. Exp. Immunol. 143, 427–434. doi: 10.1111/j.1365-2249.2006.03015.x, PMID: 16487241 PMC1809617

[ref93] Matundan H. H. Sin J. Rivas M. N. Fishbein M. C. Lehman T. J. Chen S.. (2019). Myocardial fibrosis after adrenergic stimulation as a long-term sequela in a mouse model of Kawasaki disease vasculitis. JCI Insight 4:279. doi: 10.1172/jci.insight.126279, PMID: 30728329 PMC6413776

[ref94] McCrindle B. W. Rowley A. H. Newburger J. W. Burns J. C. Bolger A. F. Gewitz M.. (2017). Diagnosis, treatment, and long-term Management of Kawasaki Disease: a scientific statement for health professionals from the American Heart Association. Circulation 135, e927–e999. doi: 10.1161/CIR.0000000000000484, PMID: 28356445

[ref95] Miyata I. Kato A. Ouchi K. (2022). Evaluation of anti-Yersinia psueudotuberculosis-derived mitogen antibody in intravenous immunoglobulin products. J. Infect. Chemother. 28, 1582–1583. doi: 10.1016/j.jiac.2022.07.020, PMID: 35934232

[ref96] Mohandas S. Jagannathan P. Henrich T. J. Sherif Z. A. Bime C. Quinlan E.. (2023). Immune mechanisms underlying COVID-19 pathology and post-acute sequelae of SARS-CoV-2 infection (PASC). Elife 12:14. doi: 10.7554/eLife.86014, PMID: 37233729 PMC10219649

[ref97] Moynahan E. J. (1987). Kawasaki-disease—a novel feline virus transmitted by fleas. Lancet 1:195. PMID: 2880020 10.1016/s0140-6736(87)90006-7

[ref98] Murata H. (1979). Experimental candida-induced arteritis in mice-relation to arteritis in the mucocutaneous lymph-node syndrome. Microbiol. Immunol. 23, 825–831. doi: 10.1111/j.1348-0421.1979.tb02815.x, PMID: 395420

[ref99] Nadig P. L. Joshi V. Pilania R. K. Kumrah R. Kabeerdoss J. Sharma S.. (2023). Intravenous immunoglobulin in Kawasaki disease-evolution and pathogenic mechanisms. Diagnostics 13:338. doi: 10.3390/diagnostics13142338, PMID: 37510082 PMC10378342

[ref100] Nagata S. Yamashiro Y. Ohtsuka Y. Shimizu T. Sakurai Y. Misawa S.. (2009). Heat shock proteins and superantigenic properties of bacteria from the gastrointestinal tract of patients with Kawasaki disease. Immunology 128, 511–520. doi: 10.1111/j.1365-2567.2009.03135.x, PMID: 19950419 PMC2792135

[ref101] Nagi-Miura N. Adachi Y. Ohno N. (2008). Coronary arteritis induced by CAWS (*Candida albicans* water-soluble fraction) in various strains of mice. Nippon Ishinkin Gakkai Zasshi 49, 287–292. doi: 10.3314/jjmm.49.287, PMID: 19001755

[ref102] Nagi-Miura N. Shingo Y. Adachi Y. Ishida-Okawara A. Oharaseki T. Takahashi K.. (2004). Induction of coronary arteritis with administration of CAWS (*Candida albicans* water-soluble fraction) depending on mouse strains. Immunopharmacol. Immunotoxicol. 26, 527–543. doi: 10.1081/iph-200042295, PMID: 15658603

[ref103] Nakamura T. Yamamura J.-I. Sato H. Kakinuma H. Takahashi H. (2007). Vasculitis induced by immunization with Bacillus Calmette-Guerin followed by atypical mycobacterium antigen: a new mouse model for Kawasaki disease. FEMS Immunol. Med. Microbiol. 49, 391–397. doi: 10.1111/j.1574-695X.2007.00217.x, PMID: 17298582 PMC7110316

[ref104] Nakamura J. Watanabe S. Kimura H. Kobayashi M. Karasawa T. Kamata R.. (2018). Adeno-associated virus vector-mediated Interleukin-10 induction prevents vascular inflammation in a murine model of Kawasaki disease. Sci. Rep. 8:7601. doi: 10.1038/s41598-018-25856-0, PMID: 29765083 PMC5953966

[ref105] Nakamura Y. Yashiro M. Uehara R. Oki I. Watanabe M. Yanagawa H. (2008). Epidemiologic features of Kawasaki disease in Japan: results from the nationwide survey in 2005-2006. J. Epidemiol. 18, 167–172. doi: 10.2188/jea.je2008001, PMID: 18635901 PMC4771586

[ref106] Nakamura Y. Yashiro M. Uehara R. Sadakane A. Tsuboi S. Aoyama Y.. (2012). Epidemiologic features of Kawasaki disease in Japan: results of the 2009-2010 nationwide survey. J. Epidemiol. 22, 216–221. doi: 10.2188/jea.je20110126, PMID: 22447211 PMC3798622

[ref107] Nigro G. Zerbini M. Krzysztofiak A. Gentilomi G. Porcaro M. A. Mango T.. (1994). Active or recent parvovirus B19 infection in children with Kawasaki disease. Lancet 343, 1260–1261. doi: 10.1016/s0140-6736(94)92154-7 7910278

[ref108] Noval Rivas M. Lee Y. Wakita D. Chiba N. Dagvadorj J. Shimada K.. (2017). CD8+ T cells contribute to the development of coronary arteritis in the *Lactobacillus casei* Cell Wall extract-induced murine model of Kawasaki disease. Arthritis Rheumatol. 69, 410–421. doi: 10.1002/art.39939, PMID: 27696768 PMC5274597

[ref109] Noval Rivas M. Arditi M. (2020). Kawasaki disease: pathophysiology and insights from mouse models. Nat. Rev. Rheumatol. 16, 391–405. doi: 10.1038/s41584-020-0426-0, PMID: 32457494 PMC7250272

[ref110] Numazaki K. Chiba S. (1996). Kawasaki disease and *Chlamydia Pneumoniae* infection. J. Infect. Chemother. 2, 264–265. doi: 10.1007/bf02355125 29681378

[ref111] Ocho K. Iwamuro M. Hasegawa K. Hagiya H. Rai K. Yumoto T.. (2018). Far East scarlet-like fever masquerading as adult-onset Kawasaki disease. Intern. Med. 57, 437–440. doi: 10.2169/internalmedicine.9250-17, PMID: 29093407 PMC5827330

[ref112] Oharaseki T. Yokouchi Y. Enomoto Y. Sato W. Ishibashi K. Miura N.. (2020). Recognition of alpha-mannan by dectin 2 is essential for onset of Kawasaki disease-like murine vasculitis induced by *Candida albicans* cell-wall polysaccharide. Mod. Rheumatol. 30, 350–357. doi: 10.1080/14397595.2019.1601852, PMID: 30924376

[ref113] Ohashi R. Fukazawa R. Watanabe M. Tajima H. Nagi-Miura N. Ohno N.. (2013). Etanercept suppresses arteritis in a murine model of Kawasaki disease: a comparative study involving different biological agents. J. Vasc. Med. 2013:543141. doi: 10.1155/2013/543141, PMID: 23606968 PMC3626397

[ref114] Ohnishi T. Nakazawa M. Wada N. Abe J. Kamimaki I. (2022). *Yersinia pseudotuberculosis* infection accompanied by intussusception and incomplete Kawasaki disease in a 7-year-old girl. Keio J. Med. 71, 50–52. doi: 10.2302/kjm.2021-0002-CR, PMID: 34108299

[ref115] Okano M. Thiele G. M. Sakiyama Y. Matsumoto S. Purtilo D. T. (1990). Adenovirus infection in patients with Kawasaki disease. J. Med. Virol. 32, 53–57. doi: 10.1002/jmv.1890320109 2173738

[ref116] Ouldali N. Pouletty M. Mariani P. Beyler C. Blachier A. Bonacorsi S.. (2020). Emergence of Kawasaki disease related to SARS-CoV-2 infection in an epicentre of the French COVID-19 epidemic: a time-series analysis. Lancet Child Adolesc Health 4, 662–668. doi: 10.1016/S2352-4642(20)30175-9, PMID: 32622376 PMC7332278

[ref117] Oura K. Ishikawa S. Shiraishi H. Maruo Y. Sato N. Suganuma T.. (2022). A one-year-old girl with human parvovirus B19 infection and Hypocomplementemia mimicking incomplete Kawasaki disease. J Med Cases 13, 229–234. doi: 10.14740/jmc3917, PMID: 35655625 PMC9119372

[ref118] Ozeki Y. Yamada F. Kishimoto T. Yashiro M. Nakamura Y. (2017). Epidemiologic features of Kawasaki disease: winter versus summer. Pediatr. Int. 59, 821–825. doi: 10.1111/ped.13293 28387988

[ref119] Ozeki Y. Yamada F. Saito A. Kishimoto T. Yashiro M. Makino N.. (2018). Epidemiologic features of Kawasaki disease distinguished by seasonal variation: an age-specific analysis. Ann. Epidemiol. 28, 796–800. doi: 10.1016/j.annepidem.2018.08.004, PMID: 30181018

[ref120] Paniz-Mondolfi A. E. van den Akker T. Marquez-Colmenarez M. C. Delgado-Noguera L. A. Valderrama O. Sordillo E. M. (2020). Kawasaki disease seasonality in Venezuela supports an arbovirus infection trigger. J. Med. Virol. 92, 2903–2910. doi: 10.1002/jmv.26381, PMID: 32740967

[ref121] Principi N. Rigante D. Esposito S. (2013). The role of infection in Kawasaki syndrome. J. Infect. 67, 1–10. doi: 10.1016/j.jinf.2013.04.004 23603251 PMC7132405

[ref122] Raut S. Roychowdhoury S. Bhakta S. Sarkar M. Nandi M. (2021). Incomplete Kawasaki disease as presentation of COVID-19 infection in an infant: a case report. J. Trop. Pediatr. 67:47. doi: 10.1093/tropej/fmaa047, PMID: 32756980 PMC7454926

[ref123] Rehman S. Majeed T. Ansari M. A. Al-Suhaimi E. A. (2020). Syndrome resembling Kawasaki disease in COVID-19 asymptomatic children. J. Infect. Public Health 13, 1830–1832. doi: 10.1016/j.jiph.2020.08.003 32919931 PMC7439985

[ref124] Rigante D. Cantarini L. Piastra M. Angelone D. F. Valentini P. Pardeo M.. (2012). Kawasaki syndrome and concurrent Coxsackie virus B3 infection. Rheumatol. Int. 32, 4037–4040. doi: 10.1007/s00296-010-1613-0, PMID: 21052673 PMC7080020

[ref125] Rivera-Figueroa E. I. Santos R. Simpson S. Garg P. (2020). Incomplete Kawasaki disease in a child with COVID-19. Indian Pediatr. 57, 680–681. doi: 10.1007/s13312-020-1900-0, PMID: 32393680 PMC7387257

[ref126] Rodo X. Ballester J. Cayan D. Melish M. E. Nakamura Y. Uehara R.. (2011). Association of Kawasaki disease with tropospheric wind patterns. Sci. Rep. 1:152. doi: 10.1038/srep00152, PMID: 22355668 PMC3240972

[ref127] Rodo X. Curcoll R. Robinson M. Ballester J. Burns J. C. Cayan D. R.. (2014). Tropospheric winds from northeastern China carry the etiologic agent of Kawasaki disease from its source to Japan. Proc. Natl. Acad. Sci. U. S. A. 111, 7952–7957. doi: 10.1073/pnas.1400380111, PMID: 24843117 PMC4050536

[ref128] Roe K. (2020). A viral infection explanation for Kawasaki disease in general and for COVID-19 virus-related Kawasaki disease symptoms. Inflammopharmacology 28, 1219–1222. doi: 10.1007/s10787-020-00739-x, PMID: 32638151 PMC7340733

[ref129] Rosenfeld N. Tasher D. Ovadia A. Abiri S. Dalal I. (2020). Kawasaki disease with a concomitant primary Epstein—Barr virus infection. Pediatr. Rheumatol. Online J. 18:65. doi: 10.1186/s12969-020-00459-0, PMID: 32787862 PMC7425362

[ref130] Rosenkranz M. E. Schulte D. J. Agle L. M. Wong M. H. Zhang W. Ivashkiv L.. (2005). TLR2 and MyD88 contribute to *Lactobacillus casei* extract-induced focal coronary arteritis in a mouse model of Kawasaki disease. Circulation 112, 2966–2973. doi: 10.1161/CIRCULATIONAHA.105.537530, PMID: 16275884

[ref131] Rowley A. H. Baker S. C. Shulman S. T. Rand K. H. Tretiakova M. S. Perlman E. J.. (2011). Ultrastructural, immunofluorescence, and RNA evidence support the hypothesis of a "new" virus associated with Kawasaki disease. J Infect Dis 203, 1021–1030. doi: 10.1093/infdis/jiq136 21402552 PMC3068030

[ref132] Sancho-Shimizu V. Brodin P. Cobat A. Biggs C. M. Toubiana J. Lucas C. L.. (2021). SARS-CoV-2-related MIS-C: a key to the viral and genetic causes of Kawasaki disease? J. Exp. Med. 218:446. doi: 10.1084/jem.20210446, PMID: 33904890 PMC8080850

[ref133] Sandhaus H. Crosby D. Sharma A. Gregory S. R. (2020). Association between COVID-19 and Kawasaki disease: vigilance required from otolaryngologists. Otolaryngol. Head Neck Surg. 163, 316–317. doi: 10.1177/0194599820930238, PMID: 32423291

[ref134] Santos R. A. Nogueira C. S. Granja S. Baptista J. B. Ribeiro M. L. Rocha M. G. (2011). Kawasaki disease and human bocavirus--potential association? J. Microbiol. Immunol. Infect. 44, 235–237. doi: 10.1016/j.jmii.2011.01.016 21524620

[ref135] Schildgen O. Muller A. Allander T. Mackay I. M. Volz S. Kupfer B.. (2008). Human bocavirus: passenger or pathogen in acute respiratory tract infections? Clin. Microbiol. Rev. 21:291-+. doi: 10.1128/cmr.00030-07, PMID: 18400798 PMC2292574

[ref136] Schnaar D. A. Bell D. M. (1982). Kawasaki syndrome in two cousins with parainfluenza virus infection. Am. J. Dis. Child. 136, 554–555. doi: 10.1001/archpedi.1982.03970420078019 6283878

[ref137] Sharma C. Ganigara M. Galeotti C. Burns J. Berganza F. M. Hayes D. A.. (2021). Multisystem inflammatory syndrome in children and Kawasaki disease: a critical comparison. Nat. Rev. Rheumatol. 17, 731–748. doi: 10.1038/s41584-021-00709-9, PMID: 34716418 PMC8554518

[ref138] Shike H. Shimizu C. Kanegaye J. T. Foley J. L. Schnurr D. P. Wold L. J.. (2005). Adenovirus, adeno-associated virus and Kawasaki disease. Pediatr. Infect. Dis. J. 24, 1011–1014. doi: 10.1097/01.inf.0000183769.31951.1e, PMID: 16282942

[ref139] Shimizu C. Shike H. Baker S. C. Garcia F. van der Hoek L. Kuijpers T. W.. (2005). Human coronavirus NL63 is not detected in the respiratory tracts of children with acute Kawasaki disease. J Infect Dis 192, 1767–1771. doi: 10.1086/497170, PMID: 16235175 PMC2888540

[ref140] Shirato K. Imada Y. Kawase M. Nakagaki K. Matsuyama S. Taguchi F. (2014). Possible involvement of infection with human coronavirus 229E, but not NL63, in Kawasaki disease. J. Med. Virol. 86, 2146–2153. doi: 10.1002/jmv.23950, PMID: 24760654 PMC7166330

[ref141] Shulman S. T. Rowley A. H. (2015). Kawasaki disease: insights into pathogenesis and approaches to treatment. Nat. Rev. Rheumatol. 11, 475–482. doi: 10.1038/nrrheum.2015.54, PMID: 25907703

[ref142] Sokolovsky S. Soni P. Hoffman T. Kahn P. Scheers-Masters J. (2021). COVID-19 associated Kawasaki-like multisystem inflammatory disease in an adult. Am. J. Emerg. Med. 39, 253.e1–253.e2. doi: 10.1016/j.ajem.2020.06.053, PMID: 32631771 PMC7315983

[ref143] Sopontammarak S. Promphan W. Roymanee S. Phetpisan S. (2008). Positive serology for dengue viral infection in pediatric patients with Kawasaki disease in southern Thailand. Circ. J. 72, 1492–1494. doi: 10.1253/circj.CJ-08-0158, PMID: 18724028

[ref144] Sosa T. Brower L. Divanovic A. (2019). Diagnosis and Management of Kawasaki Disease. JAMA Pediatr. 173, 278–279. doi: 10.1001/jamapediatrics.2018.3307 30667467

[ref145] Spezia P. G. Filippini F. Nagao Y. Sano T. Ishida T. Maggi F. (2023a). Identification of Torquetenovirus species in patients with Kawasaki disease using a newly developed species-specific PCR method. Int. J. Mol. Sci. 24:674. doi: 10.3390/ijms24108674, PMID: 37240024 PMC10218515

[ref146] Spezia P. G. Matsudaira K. Filippini F. Miyamura T. Okada K. Nagao Y.. (2023b). Viral load of Torquetenovirus correlates with Sano's score and levels of total bilirubin and aspartate aminotransferase in Kawasaki disease. Sci. Rep. 13:18033. doi: 10.1038/s41598-023-45327-5, PMID: 37865714 PMC10590372

[ref147] Stock A. T. Hansen J. A. Sleeman M. A. McKenzie B. S. Wicks I. P. (2016). GM-CSF primes cardiac inflammation in a mouse model of Kawasaki disease. J. Exp. Med. 213, 1983–1998. doi: 10.1084/jem.20151853, PMID: 27595596 PMC5030799

[ref148] Stower H. (2020). Kawasaki disease in a COVID-19-struck region. Nat. Med. 26:822. doi: 10.1038/s41591-020-0959-4, PMID: 32528149

[ref149] Strigl S. Kutlin A. Roblin P. M. Shulman S. Hammerschlag M. R. (2000). Is there an association between Kawasaki disease and *Chlamydia pneumoniae*? J Infect Dis 181, 2103–2105. doi: 10.1086/315526 10837204

[ref150] Suganuma E. Sato S. Honda S. Nakazawa A. (2020). A novel mouse model of coronary stenosis mimicking Kawasaki disease induced by *Lactobacillus casei* cell wall extract. Exp. Anim. 69, 233–241. doi: 10.1538/expanim.19-0124 31932543 PMC7220718

[ref151] Tabata A. Ohkuni H. Itoh Y. Fukunaga Y. Tomoyasu T. Nagamune H. (2021). Complete genome sequence of *Streptococcus mitis* strain Nm-65, isolated from a patient with Kawasaki disease. Microbiol Resourc Announce 10:20. doi: 10.1128/mra.01239-20, PMID: 33414340 PMC8407716

[ref152] Tada R. Nagi-Miura N. Adachi Y. Ohno N. (2008). The influence of culture conditions on vasculitis and anaphylactoid shock induced by fungal pathogen *Candida albicans* cell wall extract in mice. Microb. Pathog. 44, 379–388. doi: 10.1016/j.micpath.2007.10.013, PMID: 18065191

[ref153] Tada R. Yamanaka D. Nagi-Miura N. Adachi Y. Ohno N. (2014). Vasculitis and Anaphylactoid shock induced in mice by Cell Wall extract of the fungus Candida metapsilosis. Pol. J. Microbiol. 63, 223–230. doi: 10.33073/pjm-2014-029, PMID: 25115117

[ref154] Tahara M. Baba K. Waki K. Arakaki Y. (2006). Analysis of Kawasaki disease showing elevated antibody titres of *Yersinia pseudotuberculosis*. Acta Paediatr. 95, 1661–1664. doi: 10.1080/08035250600750080 17129979

[ref155] Takahashi K. Oharaseki T. Wakayama M. Yokouchi Y. Naoe S. Murata H. (2004). Histopathological features of murine systemic vasculitis caused by *Candida albicans* extract—an animal model of Kawasaki disease. Inflamm. Res. 53, 72–77. doi: 10.1007/s00011-003-1225-1, PMID: 15021972

[ref156] Takahashi K. Oharaseki T. Yokouchi Y. Hiruta N. Naoe S. (2010a). Kawasaki disease as a systemic vasculitis in childhood. Ann. Vasc. Dis. 3, 173–181. doi: 10.3400/avd.sasvp01003, PMID: 23555407 PMC3595783

[ref157] Takahashi K. Oharaseki T. Yokouchi Y. Miura N. N. Ohno N. Okawara A. I.. (2010b). Administration of human immunoglobulin suppresses development of murine systemic vasculitis induced with *Candida albicans* water-soluble fraction: an animal model of Kawasaki disease. Mod. Rheumatol. 20, 160–167. doi: 10.1007/s10165-009-0250-5, PMID: 19943075

[ref158] Takemoto R. Suzuki T. Hashiguchi T. Yanagi Y. Shirogane Y. (2022). Short-stalk isoforms of CADM1 and CADM2 trigger Neuropathogenic measles virus-mediated membrane fusion by interacting with the viral hemagglutinin. J. Virol. 96:e0194921. doi: 10.1128/jvi.01949-21, PMID: 34788082 PMC8826817

[ref159] Takeshita S. Kobayashi I. Kawamura Y. Tokutomi T. Sekine I. (2002). Characteristic profile of intestinal microflora in Kawasaki disease. Acta Paediatr. 91, 783–788. doi: 10.1080/08035250213221, PMID: 12200903

[ref160] Tanaka H. Yanai C. Miura N. N. Ishibashi K.-I. Yamanaka D. Ohnishi H.. (2020). Coronary Vasculitis induced in mice by Cell Wall Mannoprotein fractions of clinically isolated Candida species. Med Mycol J 61, 33–48. doi: 10.3314/mmj.20-00008, PMID: 32863327

[ref161] Tang Y. Yan W. Sun L. Huang J. Qian W. Hou M.. (2016). Kawasaki disease associated with *Mycoplasma pneumoniae*. Ital. J. Pediatr. 42:83. doi: 10.1186/s13052-016-0292-1, PMID: 27609267 PMC5016862

[ref162] Tasaka K. Hamashima Y. (1978). Studies on rickettsia-like body in Kawasaki disease. Attempts of the isolation and characterization. Acta Pathol. Jpn. 28, 235–245. doi: 10.1111/j.1440-1827.1978.tb00535.x, PMID: 354323

[ref163] Thissen J. B. Isshiki M. Jaing C. Nagao Y. Lebron Aldea D. Allen J. E.. (2018). A novel variant of torque Teno virus 7 identified in patients with Kawasaki disease. PloS One 13:e0209683. doi: 10.1371/journal.pone.0209683, PMID: 30592753 PMC6310298

[ref164] Tomita S. Kato H. Fujimoto T. Inoue O. Koga Y. Kuriya N. (1987). Cytopathogenic protein in filtrates from cultures of *propionibacterium acnes* isolated from patients with Kawasaki-disease. Br. Med. J. 295, 1229–1232. doi: 10.1136/bmj.295.6608.1229, PMID: 3120957 PMC1248303

[ref165] Toprak D. Serce O. Turel O. Atici S. Soysal A. Bakir M. (2015). Is varicella zoster virus an etiologic factor in Kawasaki disease? A case report and review of the literature. Glob Pediatr Health 2, 2333794X14567194–12333794X14567194. doi: 10.1177/2333794x14567194, PMID: 27335935 PMC4784607

[ref166] Toubiana J. Poirault C. Corsia A. Bajolle F. Fourgeaud J. Angoulvant F.. (2020). Kawasaki-like multisystem inflammatory syndrome in children during the covid-19 pandemic in Paris, France: prospective observational study. BMJ 369:m2094. doi: 10.1136/bmj.m2094, PMID: 32493739 PMC7500538

[ref167] Tsurumizu T. Okonogi H. Shibusawa T. Hashimoto T. Makino M. Ota H.. (1991). A case of Kawasaki's disease combined with septicemia--isolation of Streptococcus sanguis (MCLS-1) and *Streptococcus pyogenes* from blood at the acute stage. *Kansenshogaku zasshi*. J Japan Assoc Infect Dis 65, 124–128. 10.11150/kansenshogakuzasshi1970.65.124 2066585

[ref168] Turkay S. Odemis E. Karadag A. (2006). Kawasaki disease onset during concomitant infections with varicella zoster and Epstein-Barr virus. J. Natl. Med. Assoc. 98, 1350–1352. 16916136 PMC2569579

[ref169] Ueda Y. Kenzaka T. Noda A. Yamamoto Y. Matsumura M. (2015). Adult-onset Kawasaki disease (mucocutaneous lymph node syndrome) and concurrent Coxsackievirus A4 infection: a case report. Int Med Case Rep J 8, 225–230. doi: 10.2147/IMCRJ.S90685, PMID: 26491373 PMC4599061

[ref170] Umezawa T. Saji T. Matsuo N. Odagiri K. (1989). Chest-x-ray findings in the acute phase of Kawasaki disease. Pediatr. Radiol. 20, 48–51. doi: 10.1007/bf02010633 2602015

[ref171] Valtuille Z. Lefevre-Utile A. Ouldali N. Beyler C. Boizeau P. Dumaine C.. (2023). Calculating the fraction of Kawasaki disease potentially attributable to seasonal pathogens: a time series analysis. EClinicalMedicine 61:102078. doi: 10.1016/j.eclinm.2023.102078, PMID: 37483549 PMC10359724

[ref172] van Stijn D. Slegers A. Zaaijer H. Kuijpers T. (2020). Lower CMV and EBV exposure in children with Kawasaki disease suggests an under-challenged immune system. Front. Pediatr. 8:627957. doi: 10.3389/fped.2020.627957, PMID: 33585370 PMC7873854

[ref173] Ventura M. J. Guajardo E. Clark E. H. Bhairavarasu K. Kherallah R. Y. DiNardo A. R.. (2020). Correspondence on 'Paediatric multisystem inflammatory syndrome temporally associated with SARS-CoV-2 mimicking Kawasaki disease (Kawa-COVID-19): a multicentre cohort' by Pouletty et al. Ann. Rheum. Dis. 81:e239. doi: 10.1136/annrheumdis-2020-218959 32978236

[ref174] Verma N. A. Zheng X. T. Harris M. U. Cadichon S. B. Melin-Aldana H. Khetsuriani N.. (2009). Outbreak of life-threatening coxsackievirus B1 myocarditis in neonates. Clin. Infect. Dis. 49, 759–763. doi: 10.1086/605089, PMID: 19622042

[ref175] Vincent P. Salo E. Skurnik M. Fukushima H. Simonet M. (2007). Similarities of Kawasaki disease and *Yersinia pseudotuberculosis* infection epidemiology. Pediatr. Infect. Dis. J. 26, 629–631. doi: 10.1097/INF.0b013e3180616d3c, PMID: 17596806

[ref176] Viner R. M. Whittaker E. (2020). Kawasaki-like disease: emerging complication during the COVID-19 pandemic. Lancet 395, 1741–1743. doi: 10.1016/S0140-6736(20)31129-6, PMID: 32410759 PMC7220168

[ref177] Wang C.-Y. Song C.-M. Liu G.-H. Zhang H. Chen F.-S. Lin H. (2021). Association between *Mycoplasma pneumoniae* infection and coronary artery aneurysm in children with Kawasaki disease. Iran. J. Pediatr. 31:737. doi: 10.5812/ijp.104737

[ref178] Wang J. Sun F. Deng H.-L. Liu R.-Q. (2019). Influenza a (H1N1) pdm09 virus infection in a patient with incomplete Kawasaki disease a case report. Medicine 98:e15009. doi: 10.1097/md.0000000000015009, PMID: 30985646 PMC6485757

[ref179] Wang H. Xia Y. Fu S. Wang W. Xie C. Zhang Y.. (2016). Notch4 signaling pathway of endothelial progenitor cells in a Kawasaki disease model induced by *Lactobacillus casei* Cell Wall extract. J. Vasc. Res. 53, 340–348. doi: 10.1159/000449061 28013300

[ref180] Wann E. R. Fehringer A. P. Ezepchuk Y. V. Schlievert P. M. Bina P. Reiser R. F.. (1999). *Staphylococcus aureus* isolates from patients with Kawasaki disease express high levels of protein a. Infect. Immun. 67, 4737–4743. doi: 10.1128/IAI.67.9.4737-4743.1999, PMID: 10456925 PMC96803

[ref181] Weng K.-P. Wei J. C.-C. Hung Y.-M. Huang S.-H. Chien K.-J. Lin C.-C.. (2018). Enterovirus infection and subsequent risk of Kawasaki disease: a population-based cohort study. Pediatr. Infect. Dis. J. 37, 310–315. doi: 10.1097/inf.0000000000001748 28834956

[ref182] Whitby D. Hoad J. G. Tizard E. J. Dillon M. J. Weber J. N. Weiss R. A.. (1991). Isolation of measles-virus from child with Kawasaki-disease. Lancet 338:1215. doi: 10.1016/0140-6736(91)92085-g, PMID: 1682629

[ref183] Xiao H. Hu B. Luo R. Hu H. Zhang J. Kuang W.. (2020). Chronic active Epstein-Barr virus infection manifesting as coronary artery aneurysm and uveitis. Virol. J. 17:8. doi: 10.1186/s12985-020-01409-8, PMID: 33121509 PMC7597064

[ref184] Xie L. P. Yan W. L. Huang M. Huang M. R. Chen S. Huang G. Y.. (2020). Epidemiologic features of Kawasaki disease in Shanghai from 2013 through 2017. J. Epidemiol. 30, 429–435. doi: 10.2188/jea.JE20190065, PMID: 31548437 PMC7492704

[ref185] Yamada H. Ohta H. Hasegawa S. Azuma Y. Hasegawa M. Kadoya R.. (2016). Two infants with tuberculid associated with Kawasaki disease. Hum. Vaccin. Immunother. 12, 2772–2776. doi: 10.1080/21645515.2016.1208329, PMID: 27435523 PMC5137533

[ref186] Yamashiro Y. Nagata S. Ohtsuka Y. Oguchi S. Shimizu T. (1996). Microbiologic studies on the small intestine in Kawasaki disease. Pediatr. Res. 39, 622–624. doi: 10.1203/00006450-199604000-00010, PMID: 8848335

[ref187] Yanai C. Tanaka H. Miura N. N. Ishibashi K.-I. Yamanaka D. Ohnishi H.. (2020). Coronary Vasculitis induced in mice by the Cell Wall Mannoprotein of *Candida krusei*. Biol. Pharm. Bull. 43, 848–858. doi: 10.1248/bpb.b19-01060, PMID: 32161223

[ref188] Yoshikane Y. Koga M. Imanaka-Yoshida K. Cho T. Yamamoto Y. Yoshida T.. (2015). JNK is critical for the development of *Candida albicans*-induced vascular lesions in a mouse model of Kawasaki disease. Cardiovasc. Pathol. 24, 33–40. doi: 10.1016/j.carpath.2014.08.005 25242023

[ref189] Zhang Q. Y. Xu B. W. Du J. B. (2021). Similarities and differences between multiple inflammatory syndrome in children associated with COVID-19 and Kawasaki disease: clinical presentations, diagnosis, and treatment. World J. Pediatr. 17, 335–340. doi: 10.1007/s12519-021-00435-y, PMID: 34013488 PMC8134825

